# A universal material model subroutine for soft matter systems

**DOI:** 10.1007/s00366-024-02031-w

**Published:** 2024-09-18

**Authors:** Mathias Peirlinck, Juan A. Hurtado, Manuel K. Rausch, Adrián Buganza Tepole, Ellen Kuhl

**Affiliations:** 1 Department of BioMechanical Engineering, Faculty of Mechanical Engineering, Delft University of Technology, Delft, the Netherlands; 2 Dassault Systèmes, Providence, RI USA; 3 Department of Mechanical Engineering, University of Texas at Austin, Austin, TX USA; 4 Department of Mechanical Engineering, Purdue University, West Lafayette, IN USA; 5 Department of Mechanical Engineering, Stanford University, Stanford, CA USA

**Keywords:** Constitutive modeling, Finite element method, Soft matter, Material modeling, Tissue mechanics

## Abstract

Soft materials play an integral part in many aspects of modern life including autonomy, sustainability, and human health, and their accurate modeling is critical to understand their unique properties and functions. Today’s finite element analysis packages come with a set of pre-programmed material models, which may exhibit restricted validity in capturing the intricate mechanical behavior of these materials. Regrettably, incorporating a modified or novel material model in a finite element analysis package requires non-trivial in-depth knowledge of tensor algebra, continuum mechanics, and computer programming, making it a complex task that is prone to human error. Here we design a universal material subroutine, which automates the integration of novel constitutive models of varying complexity in non-linear finite element packages, with no additional analytical derivations and algorithmic implementations. We demonstrate the versatility of our approach to seamlessly integrate innovative constitutive models from the material point to the structural level through a variety of soft matter case studies: a frontal impact to the brain; reconstructive surgery of the scalp; diastolic loading of arteries and the human heart; and the dynamic closing of the tricuspid valve. Our universal material subroutine empowers all users, not solely experts, to conduct reliable engineering analysis of soft matter systems. We envision that this framework will become an indispensable instrument for continued innovation and discovery within the soft matter community at large.

## Motivation

Understanding the mechanical behavior of soft matter is pivotal across various scientific and engineering domains, ranging from biophysics, over soft robotics, to biomedical and material science engineering. Biological materials, composites, polymers, foams, and gels all exhibit complex non-linear mechanical behaviors and functions, which result from the intrinsic architecture and interactions of their constituent molecules or particles. To characterize this behavior, a multitude of constitutive material models have been proposed in the literature [1].

Finite element analysis provides a versatile and powerful framework to evaluate these highly nonlinear material models and predict their mechanical response within complex geometries and under various loading conditions. Most contemporary finite element software packages offer an extensive number of standard isotropic and anisotropic hyperelastic material models, including neo-Hooke [2], Mooney Rivlin [3, 4], Ogden [5], or Yeoh [6]. However, the implementation of newly discovered constitutive models requires the definition of novel material model subroutines or plugins, which map the computational domain’s second-order kinematic deformation gradient tensor to a second-order Cauchy stress tensor [7]. These material subroutines are evaluated within every finite element, at each integration point, within every time step, at each Newton iteration.

Unfortunately, the efficient integration of novel constitutive models into non-linear finite element software packages is a complex task [8, 9]. The user needs to derive and implement explicit forms of the second-order Cauchy stress tensor and the fourth-order spatial elasticity tensor [10]. The derivation and coding of these complicated tensorial expressions can be an extremely hard task [11], and requires a non-trivial deep understanding of tensor algebra, continuum mechanics, computational algorithms, data structures, and software architecture [12]. Non-surprisingly, such endeavors are highly subject to human errors [13]. This high degree of effort and risk of human error when integrating novel constitutive models in finite element packages limits its use to expert specialists, and, as such, hampers research progress, dissemination, and sharing of models and results amongst a broad and inclusive community.

In this work, we streamline the implementation of novel constitutive models into existing finite element analysis software, and mitigate the risk for human error. We provide a common language and framework for the computational mechanics community at large. We design a modular and universal material subroutine, which automates the incorporation of constitutive models of varying complexity in non-linear finite element analysis packages and requires no additional analytical derivations and algorithmic implementations by the user. First, we introduce the concept of constitutive neural networks, which form the architectural backbone for our universal material model. Next, we illustrate the universal material model itself, describe its internal structure through pseudocodes, and showcase how this subroutine can be effortlessly integrated and activated within finite element simulations. We provide specific examples on how existing constitutive models fit in our overarching framework, and how we can incorporate special constitutive cases that feature mixed invariant features. Finally, we showcase the flexibility of our approach to naturally integrate novel constitutive models from the material point level to the structural level through various soft matter modeling case studies: the mechanical simulation of a frontal impact to the brain, reconstructive surgery of the scalp, the diastolic loading of arteries and the human heart, and the dynamic closing of the tricuspid valve.

## Constitutive modeling

**Fig. 1:**
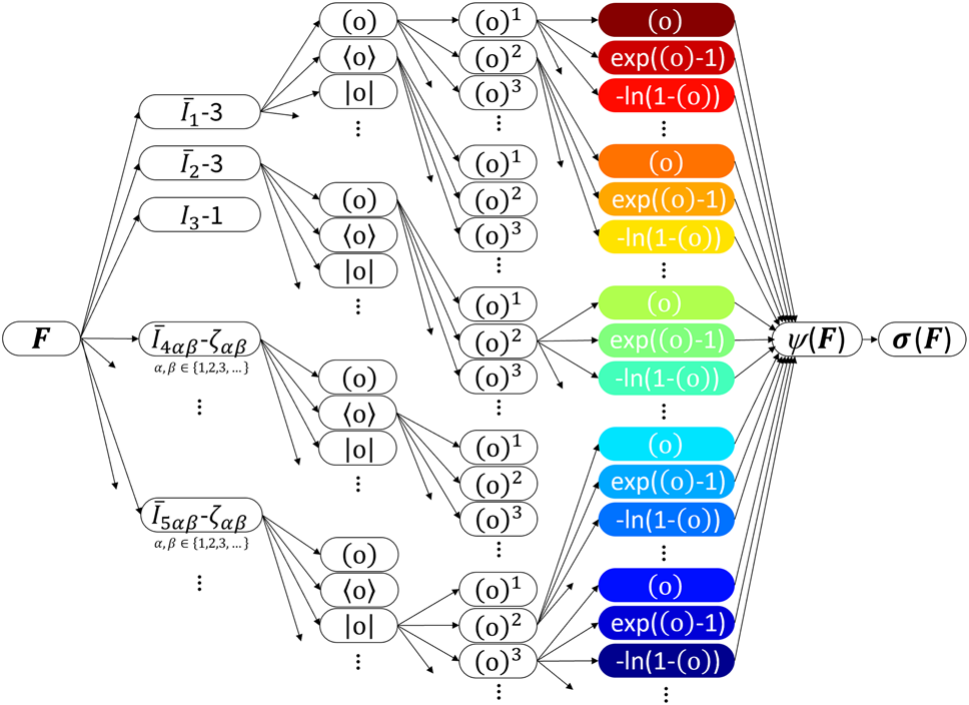
**Constitutive neural network architecture**. Anisotropic, compressible, feed forward constitutive neural network with three hidden layers to approximate the single scalar-valued free energy $\psi ({\bar{I}}_1, {\bar{I}}_2, I_3, {\bar{I}}_{{{4\alpha \beta }}},{\bar{I}}_{{{5\alpha \beta }}})$, as a function of 15 invariants of the left Cauchy-Green deformation tensor $\varvec{b}$. The zeroth layer generates identity $(\circ )$, the rectified linear unit $\langle \circ \rangle$, and the absolute value $\langle \circ \rangle$ of the 15 invariants. The first layer generates powers $(\circ )$, $(\circ )^2$, $(\circ )^3$, etc. and the second layer applies the identity $(\circ )$, the exponential $(\textrm{exp}(\circ )-1)$, and the logarithm $(-\textrm{ln}(1-(\circ )))$ to these powers. The network is not fully connected by design to satisfy the condition of polyconvexity *a priori*.

### Kinematics

We introduce the deformation map $\varvec{\varphi }$ as the mapping of material points $\varvec{X}$ in the undeformed configuration to points $\varvec{x}=\varvec{\varphi }(\varvec{X})$ in the deformed configuration [14, 15]. The gradient of the deformation map $\varvec{\varphi }$ with respect to the undeformed coordinates $\varvec{X}$ defines the deformation gradient $\varvec{F}$ with its determinant *J*,

1 $$\begin{aligned} \varvec{F} = \nabla _{{{\textbf{X}}}} \varvec{\varphi } \quad \text{ with } \quad J = \det (\varvec{F}) > 0, \end{aligned}$$

We multiplicatively decompose the deformation gradient $\varvec{F}$ into its volumetric $\varvec{F}_{{\textrm{vol}}}$ and isochoric $\bar{\varvec{F}}$ parts [16],

2 $$\begin{aligned} \varvec{F} = \varvec{F}_{{\textrm{vol}}} \cdot \bar{\varvec{F}} \quad \text{ with } \quad \varvec{F}_{{\textrm{vol}}} = J^{\frac{1}{3}}\varvec{I} \quad \text{ and } \quad \bar{\varvec{F}} = J^{-\frac{1}{3}}\varvec{F}, \end{aligned}$$

where $\cdot$ denotes the tensor product between two second order tensors. As deformation measures, we introduce the left and right Cauchy-Green deformation tensors, $\varvec{b}$ and $\varvec{C}$, and their isochoric counterparts, $\bar{\varvec{b}}$ and $\bar{\varvec{C}}$,

3 $$\begin{aligned} \begin{array}{lclcl} \varvec{b} &{}=&{} \varvec{F} &{}\cdot &{} \varvec{F}^{{\textrm{t}}} \\ \varvec{C} &{}=&{} \varvec{F}^{{\textrm{t}}} &{}\cdot &{} \varvec{F} \end{array} \qquad \text{ and } \qquad \begin{array}{lclcl} \bar{\varvec{b}} &{}=&{} \bar{\varvec{F}} &{}\cdot &{}\bar{\varvec{F}}^{{\textrm{t}}}\\ \bar{\varvec{C}} &{}=&{}\bar{\varvec{F}}^{{\textrm{t}}} &{}\cdot &{}\bar{\varvec{F}}. \end{array} \end{aligned}$$

We further assume directionally-dependent behavior, with three preferred directions, $\varvec{n}_{1}^0$, $\varvec{n}_{2}^0$, $\varvec{n}_{3}^0$, associated with the material’s internal fiber directions in the reference configuration, where all three vectors are unit vectors, $||\,\varvec{n}_{1}^0\,|| = 1$, $||\,\varvec{n}_{2}^0\,|| = 1$, $||\,\varvec{n}_{3}^0\,|| = 1$. Based on the volumetric and isochoric decomposition, and the underlying fiber orientations in the material, we characterize the deformation in terms of 15 invariants [17, 18]. More specifically, we define one isotropic volumetric invariant,

4 $$\begin{aligned} I_3 = \det (\varvec{F}^{{\textrm{t}}} \cdot \varvec{F}) = J^2 , \end{aligned}$$

two isotropic deviatoric invariants,

5 $$\begin{aligned} \begin{aligned}&{\bar{I}}_1 = [\bar{\varvec{F}}^{{\textrm{t}}} \cdot \bar{\varvec{F}} ]: \varvec{I} \\&{\bar{I}}_2 = {\frac{1}{2}} \; [ {\bar{I}}_1^2 - [\bar{\varvec{F}}^{{\textrm{t}}} \cdot \bar{\varvec{F}} ]: [\bar{\varvec{F}}^{{\textrm{t}}} \cdot \bar{\varvec{F}} ] ],\\ \end{aligned} \end{aligned}$$

six anisotropic deviatoric invariants,

6 $$\begin{aligned} \begin{array}{ll} {\bar{I}}_{{{4(11)}}} = [\bar{\varvec{F}}^{{\textrm{t}}} \cdot \bar{\varvec{F}} ]: [\varvec{n}_{1}^0 \otimes \varvec{n}_{1}^0] &\quad {\bar{I}}_{{{5(11)}}} = [\bar{\varvec{F}}^{{\textrm{t}}} \cdot \bar{\varvec{F}} ]^2: [\varvec{n}_{1}^0 \otimes \varvec{n}_{1}^0] \\ {\bar{I}}_{{{4(22)}}} = [\bar{\varvec{F}}^{{\textrm{t}}} \cdot \bar{\varvec{F}} ]: [\varvec{n}_{2}^0 \otimes \varvec{n}_{2}^0] &\quad  {\bar{I}}_{{{5(22)}}} = [\bar{\varvec{F}}^{{\textrm{t}}} \cdot \bar{\varvec{F}} ]^2: [\varvec{n}_{2}^0 \otimes \varvec{n}_{2}^0] \\ {\bar{I}}_{{{4(33)}}} = [\bar{\varvec{F}}^{{\textrm{t}}} \cdot \bar{\varvec{F}} ]: [\varvec{n}_{3}^0 \otimes \varvec{n}_{3}^0] &\quad  {\bar{I}}_{{{5(33)}}} = [\bar{\varvec{F}}^{{\textrm{t}}} \cdot \bar{\varvec{F}} ]^2: [\varvec{n}_{3}^0 \otimes \varvec{n}_{3}^0] \end{array} \end{aligned}$$

and six deviatoric coupling invariants,

7 $$\begin{aligned} \begin{array}{ll} {\bar{I}}_{{{4(12)}}} = [\bar{\varvec{F}}^{{\textrm{t}}} \cdot \bar{\varvec{F}} ]: [\varvec{n}_{1}^0 \otimes \varvec{n}_{2}^0] &{}\quad {\bar{I}}_{{{5(12)}}} = [\bar{\varvec{F}}^{{\textrm{t}}} \cdot \bar{\varvec{F}} ]^2: [\varvec{n}_{1}^0 \otimes \varvec{n}_{2}^0] \\ {\bar{I}}_{{{4(13)}}} = [\bar{\varvec{F}}^{{\textrm{t}}} \cdot \bar{\varvec{F}} ]: [\varvec{n}_{1}^0 \otimes \varvec{n}_{3}^0] &{}\quad  {\bar{I}}_{{{5(13)}}} = [\bar{\varvec{F}}^{{\textrm{t}}} \cdot \bar{\varvec{F}} ]^2: [\varvec{n}_{1}^0 \otimes \varvec{n}_{3}^0] \\ {\bar{I}}_{{{4(23)}}} = [\bar{\varvec{F}}^{{\textrm{t}}} \cdot \bar{\varvec{F}} ]: [\varvec{n}_{2}^0 \otimes \varvec{n}_{3}^0] &{}\quad  {\bar{I}}_{{{5(23)}}} = [\bar{\varvec{F}}^{{\textrm{t}}} \cdot \bar{\varvec{F}} ]^2: [\varvec{n}_{2}^0 \otimes \varvec{n}_{3}^0] \end{array} \end{aligned}$$

where $[\bar{\varvec{F}}^{{\textrm{t}}} \cdot \bar{\varvec{F}} ]^2 = \bar{\varvec{C}}\cdot \bar{\varvec{C}}$. Note that these coupling invariants reverse their sign if one of the fiber directions changes its sign, and can therefore not be considered strictly invariant. Nevertheless, these pseudo-invariants were found to be convenient for the definition of anisotropic constitutive models [19].

### Free energy function

To ensure thermodynamic consistency, we introduce the Helmholtz free energy $\psi$ as a function of the deformation gradient $\psi =\psi \left( \varvec{F} \right)$. Assuming no dissipative energy losses within the material, and rewriting the Clausius-Duhem entropy inequality [20] following the Coleman and Noll principle [21, 22], we derive

8 $$\begin{aligned} \varvec{\sigma } =\frac{1}{J} \frac{\partial \psi \left( \varvec{F} \right) }{\partial \varvec{F}} \cdot \varvec{F}^{\textrm{t}} \end{aligned}$$

as the constitutive relation between Cauchy stress $\varvec{\sigma }$ and deformation gradient $\varvec{F}$. To guarantee that our free energy function $\psi$ satisfies *material objectivity* and *material symmetry*, we further constrain our stress responses to be functions of the invariants of the left and right Cauchy Green deformation tensors $\varvec{b}$ and $\varvec{C}$ [17, 23]. This results in the general definition of the free energy function $\psi$ as a function of the 15 invariants,

9 $$\begin{aligned} \psi \left( \varvec{F} \right) \doteq \psi \left( {\bar{I}}_1 \,, {\bar{I}}_2 \,, I_3 \,, {\bar{I}}_{{{4(\alpha \beta )}}} \,, {\bar{I}}_{{{5(\alpha \beta )}}} \right) , \end{aligned}$$

with $\alpha \, , \beta \in \{ 1,2,3 \}$ and $\beta \ge \alpha$. To account for the quasi-incompressible behavior of soft materials, we make the constitutive choice to additively decompose our free energy function $\psi$ into volumetric $\psi _{{\textrm{vol}}}$ and isochoric ${\bar{\psi }}$ parts,

10 $$\begin{aligned} \psi \doteq \psi _{{\textrm{vol}}} + {\bar{\psi }}. \end{aligned}$$

Here, we define the volumetric free energy contribution,

11 $$\begin{aligned} \psi _{{\textrm{vol}}} = \psi _3(I_3) , \end{aligned}$$

in terms of the isotropic volumetric invariant $I_3$ (Eq. (4)), and the deviatoric free energy contribution,

12 $$\begin{aligned} \begin{aligned} {\bar{\psi }} = {\bar{\psi }} \left( {\bar{I}}_1 \,, {\bar{I}}_2 \,, {\bar{I}}_{{{4(\alpha \beta )}}} \,, {\bar{I}}_{{{5(\alpha \beta )}}} \right) , \end{aligned} \end{aligned}$$

as functions of the isotropic and anisotropic deviatoric invariants from Eqs. (5), (6) and (7), with $\alpha \, , \beta \in \{ 1,2,3 \}$ and $\beta \ge \alpha$.

### Constitutive neural network

With the aim to universally model a hyperelastic history-independent soft matter material behavior, we design the modular constitutive neural network architecture depicted in Fig. 1. Leveraging our prior work on automated constitutive model discovery for isotropic [24–26], transversely isotropic [27, 28], and orthotropic [29] soft materials, we create a universal function approximator, which maps the 15 invariants ${\bar{I}}_1$, ${\bar{I}}_2$, $I_3$, ${\bar{I}}_{{{4(\alpha \beta )}}}$, ${\bar{I}}_{{{5(\alpha \beta )}}}$ of the deformation gradient $\varvec{F}$ onto the free energy function $\psi \left( \varvec{F} \right)$. The constitutive relation between the Cauchy stress $\varvec{\sigma }$ and the deformation gradient $\varvec{F}$ follows naturally from the second law of thermodynamics as the derivative of the free energy function $\psi$ with respect to the deformation gradient ***F*** according to Eq. (8). We ensure a vanishing free energy $\psi \left( \varvec{F} \right) \doteq 0$ in the reference configuration, i.e., when $\varvec{F}=\varvec{I}$, by using the invariants’ deviation from the energy-free reference state, $[{\bar{I}}_1-3]$, $[{\bar{I}}_2-3]$, $[I_3-1]$, $[{\bar{I}}_{{{4(\alpha \beta )}}}-\zeta _{\alpha \beta }]$, $[{\bar{I}}_{{{5(\alpha \beta )}}}-\zeta _{\alpha \beta }]$, as constitutive neural network input. Here, $\zeta _{\alpha \beta } = \varvec{n}_{\alpha }^0 \cdot \varvec{n}_{\beta }^0$ corrects invariants ${\bar{I}}_{{{4(\alpha \beta )}}}$ and ${\bar{I}}_{{{5(\alpha \beta )}}}$ for their values in the undeformed configuration. This correction a priori ensures a *stress-free reference configuration*. To ensure polyconvexity, we design the constitutive neural network architecture as a locally connected, rather than a fully connected, feed forward neural network. Specifically, we design the free energy function as a sum of individual polyconvex subfunctions with respect to each of the individual contributing invariants. As a result, our free energy function from Eqs. (9)–(12) can be additively decomposed into

13 $$\begin{aligned} \begin{aligned} \psi =\,&{\bar{\psi }}_1 ({\bar{I}}_1) + {\bar{\psi }}_2 ({\bar{I}}_2) + \psi _3 ({I}_3) \\&+ \sum _{\alpha =1}^N \sum _{\beta =\alpha }^{N} {\bar{\psi }}_{{4 \left( \alpha \beta \right) }} \left( {\bar{I}}_{{{4\left( \alpha \beta \right) }}}\right) \\ {}&+ \sum _{\alpha =1}^N \sum _{\beta =\alpha }^{N} {\bar{\psi }}_{{5 \left( \alpha \beta \right) }} \left( {\bar{I}}_{{{5\left( \alpha \beta \right) }}}\right) , \end{aligned} \end{aligned}$$

with $\alpha \, , \beta \in \{ 1,2,3 \}$ and $\beta \ge \alpha$. Following Eq. (8), we derive the Cauchy stress

14 $$\begin{aligned} \begin{aligned} J \, \varvec{\sigma }=\,&2 \frac{\partial {\bar{\psi }}_1}{\partial {\bar{I}}_1} \, \bar{\varvec{b}} + 2 \frac{\partial {\bar{\psi }}_2}{\partial {\bar{I}}_2} \, [ {\bar{I}}_1 \bar{\varvec{b}} - \bar{\varvec{b}}^2 ] + 2 \frac{\partial \psi _3}{\partial {I}_3} \, I_3 \varvec{I} \\&+ \sum _{\alpha =1}^N \sum _{\beta =\alpha }^{N} \frac{\partial {\bar{\psi }}_{{{4(\alpha \beta )}}}}{\partial {\bar{I}}_{{{4(\alpha \beta )}}}} \, \left[ \bar{\varvec{n}}_{\alpha } \otimes \bar{\varvec{n}}_{\beta } + \bar{\varvec{n}}_{\beta } \otimes \bar{\varvec{n}}_{\alpha } \right] \\&+ \sum _{\alpha =1}^N \sum _{\beta =\alpha }^{N} \frac{\partial {\bar{\psi }}_{{{5(\alpha \beta )}}}}{\partial {\bar{I}}_{{{5(\alpha \beta )}}}} \, \left[ \bar{\varvec{n}}_{\alpha } \otimes \bar{\varvec{b}}\bar{\varvec{n}}_{\beta } + \bar{\varvec{b}}\bar{\varvec{n}}_{\alpha } \otimes \bar{\varvec{n}}_{\beta }\right. \\&+\left. \bar{\varvec{n}}_{\beta } \otimes \bar{\varvec{b}}\bar{\varvec{n}}_{\alpha } + \bar{\varvec{b}}\bar{\varvec{n}}_{\beta } \otimes \bar{\varvec{n}}_{\beta } \right] , \end{aligned} \end{aligned}$$

where $\bar{\varvec{n}}_{\alpha } = \bar{\varvec{F}} \cdot \varvec{n}_{\alpha }^0$ and $\bar{\varvec{n}}_{\beta } = \bar{\varvec{F}} \cdot \varvec{n}_{\beta }^0$ represent the deviatoric fiber vectors in the current configuration.

Our constitutive network consists of three hidden layers with activation functions that are custom-designed to satisfy physically reasonable constitutive restrictions [14, 24]. Specifically, we select from the identity $(\circ )$, the rectified linear unit function $\langle \circ \rangle$, and the modulus function $| \circ |$ for the zeroth layer of the network, from linear $(\circ )$, quadratic $(\circ )^2$, cubic $(\circ )^3$, and higher order powers for the first layer, and from linear $(\circ )$, exponential $\exp (\circ )$, and logarithmic $\ln (\circ )$ for the second layer.

## A universal material model

To predict the quasi-static response of a system undergoing mechanical loading, a non-linear finite element analysis solver iteratively evaluates whether a proposed update to the nodal displacement field satisfies the equilibrium equations that describe the force and momentum balance within the computational domain. This evaluation requires the computation of the stress tensor and the tangent stiffness tensor as functions of the proposed update to the body’s total deformation. At each time step, at each Newton–Raphson iteration, within each element, and for each integration point, the solver evaluates the constitutive response that characterizes the functional mapping between the deformation gradient $\varvec{F}$ and the Cauchy stress tensor $\varvec{\sigma }$.

Here, we outline the algorithmic framework we developed to incorporate our universal material model within a finite element analysis framework. Specifically, we set up a user-defined material model subroutine which functionally maps the local deformation gradient $\varvec{F}$ onto the free energy function $\psi$ and computes its derivative with respect to the deformation gradient $\varvec{F}$ and the Cauchy stress tensor $\varvec{\sigma }$ using Eq. (8). Additionally, we compute the tangent stiffness tensor ${\mathbb {C}}$ to improve the accuracy, stability, and efficiency of the iterative solution technique required for an accurate prediction of the non-linear material behavior under various loading conditions. The concept of our universal material subroutine is inherently modular and generally compatible with any finite element analysis package [7, 30–33]. For illustrative purposes we implement our universal material model architecture in the Abaqus finite element analysis software suite [7] as detailed in Appendix A. We make all our code and simulation files publicly available on GitHub to support the translation of our approach to other non-linear finite element analysis solvers.

### Algorithm architecture

**Fig. 2:**
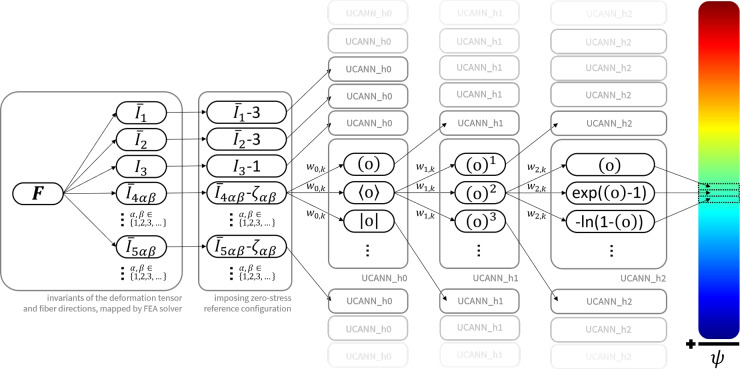
**Universal material model subroutine schematic**. Our universal material model user subroutine computes the free energy function $\psi$, its first derivatives ${\partial \psi }/{\partial {\bar{I}}_i}$, and its second derivatives ${\partial ^2 \psi }/{\partial {\bar{I}}_i \partial {\bar{I}}_j}$ with respect to the scalar invariants ${\bar{I}}_i$, derived from the deformation gradient $\varvec{F}$. These functions and derivatives are computed based on a triple set of nested activation functions $f_{0}$ (= UCANN_h0), $f_{1}$ (= UCANN_h1), and $f_{2}$ (= UCANN_h2), where each unique constitutive path forms an additive constitutive *neuron* towards the total free energy and its derivatives.

Figure 2 showcases the internal code structure of our universal material model subroutine. Our subroutine computes the free energy function $\psi$, the Cauchy stress tensor $\varvec{\sigma }$, and the tangent stiffness tensor ${\mathbb {C}}$ with respect to the scalar invariants ${\bar{I}}_i$ derived from the deformation gradient $\varvec{F}$. Following our modular constitutive neural network structure, we construct the subroutine as a triple set of nested activation functions $f_{0}$ (= uCANN_h0), $f_{1}$ (= uCANN_h1), and $f_{2}$ (= uCANN_h2). Each unique path in our constitutive neural network forms an *additive constitutive neuron contribution* to the total free energy function, the Cauchy stress tensor, and the tangent stiffness tensor, which is assembled in the overarching uCANN subroutine. Illustrative pseudocodes for each of our subroutines can be found in Appendix E. The following paragraphs summarize our adopted invariant numbering schemes and the mathematical derivations of the additive constitutive contributions to the free energy function, the Cauchy stress tensor, and the tangent stiffness tensor. We provide solver-specific integration details in Appendix A.

**Invariant numbering**

To discriminate the different scalar invariants that can be derived from the deformation gradient $\varvec{F}$ (Eqs. (4)–(7)), we adopt the invariant numbering

15 $$\begin{aligned} \begin{array}{lcl} {\bar{I}}_{1} &{}\rightarrow &{} {\bar{I}}_{\texttt {NINV}}; \; \texttt {NINV} = 1 \\ {\bar{I}}_{2} &{}\rightarrow &{} {\bar{I}}_{\texttt {NINV}}; \; \texttt {NINV} = 2 \\ I_{3} &{}\rightarrow &{} I_{\texttt {NINV}}; \; \texttt {NINV} = 3 \\ {\bar{I}}_{4(\alpha \beta )} &{}\rightarrow &{} {\bar{I}}_{\texttt {NINV}}; \; \texttt {NINV} = 4 + 2 \, (\alpha -1)+\beta \, (\beta -1) \\ {\bar{I}}_{5(\alpha \beta )} &{}\rightarrow &{} {\bar{I}}_{\texttt {NINV}}; \; \texttt {NINV} = 5 + 2 \, (\alpha -1)+\beta \, (\beta -1) \end{array} \end{aligned}$$

Dependent on the number of fiber families, this scheme automatically adapts itself to account for multiple fiber orientations. For example, when our material displays an anisotropic behavior with three families of fibers ($${\texttt {NDIR}=3}$$), there are a total of 15 invariants: $${\bar{I}}_{1}$$, $${\bar{I}}_{2}$$, $$I_{3} $$, six invariants of type $${\bar{I}}_{4(\alpha \beta )}$$, and six invariants of type $${\bar{I}}_{5(\alpha \beta )}$$, with $$\alpha \, , \beta \in \{ 1,2,3 \}$$ and $$\beta \ge \alpha $$.

**Free energy function update**

Without loss of generality, we reformulate the free energy function $\psi$ from Eq. (13) in the following form,

16 $$\begin{aligned} \begin{aligned} \psi&;= f_{2}\, {\circ}\, f_{1} \, {\circ}\, f_{0} \, ({\bar{I}}_{i}-{\bar{I}}_{i0}) \\;= \sum _{k=1}^n w_{2,k} \, f_{2,k} \, (f_{1,k} \, (f_{0,k} \,({\bar{I}}_{i,k}-{\bar{I}}_{i0,k}; w_{0,k}); w_{1,k})) , \end{aligned} \end{aligned}$$

where $f_{0}$, $f_{1}$, $f_{2}$ are the nested activation functions associated with the zeroth, first, and second layers of our modular constitutive neural network; $k=1,\ldots ,n$ defines each unique additive constitutive *neuron* that stems from the expanding nested constitutive neural network in Fig. 2; and ${\bar{I}}_{i0}$ imposes the free energy $\psi$ and Cauchy stress $\varvec{\sigma }$ to be zero in the reference configuration. As discussed above and shown in Fig. 1, these corrections amount to ${\bar{I}}_{i0}=3$ for $i=1,2$, to $I_{i0}=1$ for $i=3$, and to ${\bar{I}}_{i0}=\zeta _{\alpha \beta } = \varvec{n}_{\alpha }^0 \cdot \varvec{n}_{\beta }^0$ for $i \ge 4$ with respect to the invariant numbering scheme in Eq. (15). Our nested activation functions in Eq. (16) read

17 $$\begin{aligned} f_0 = \left\{ \begin{array}{c} {\left( \circ \right) }\\ {\langle \circ \rangle }\\ {|\circ |}\\ \vdots \end{array} \right. \; f_1 = \left\{ \begin{array}{c} {(\circ )^1}\\ {(\circ )^2}\\ {(\circ )^3}\\ \vdots \\ {(\circ )^m} \end{array} \right. \; f_2 = \left\{ \begin{array}{c} {w_1 \, (\circ )}\\ {{\textrm{exp}}(w_1 \, (\circ ))-1}\\ -{{\textrm{ln}}(1-w_1 \, (\circ ))} \\ \vdots \end{array} \right. \!\!. \end{aligned}$$

The activation function $f_0$ returns the identity, Macauley bracketed, or absolute values, $(\circ )$, $\langle \circ \rangle$, $|\circ |$ of the zero-stress reference configuration corrected invariants; $f_1$ raises these invariants to the first, second, third, or any higher order powers, $(\circ )^1, (\circ )^2, (\circ )^3, \ldots (\circ )^m$; and $f_2$ applies the identity, exponential, or natural logarithm, $(\circ )$, $(\textrm{exp}(\circ )-1)$, $(-\textrm{ln}(1-(\circ )))$, or any other thermodynamically admissible function to these powers.

**Cauchy stress tensor update**

To update the Cauchy stress tensor $\varvec{\sigma }$, we reformulate Eq. (8) in the following form,

18 $$\begin{aligned} \begin{aligned} \varvec{\sigma }&=\frac{1}{J}\frac{\partial \psi \left( \varvec{F} \right) }{\partial \varvec{F}} \cdot \varvec{F}^{{\textrm{t}}} =\sum _{k=1}^n \frac{1}{J}\frac{\partial \psi }{\partial {\bar{I}}_{i,k}}\frac{\partial {\bar{I}}_{i,k}}{\partial \varvec{F}} \cdot \varvec{F}^{{\textrm{t}}}\\&=\sum _{i} \frac{1}{J} \left( \sum _{k}^{n_i}\frac{\partial \psi }{\partial {\bar{I}}_{i,k}}\right) \frac{\partial {\bar{I}}_{i}}{\partial \varvec{F}} \cdot \varvec{F}^{{\textrm{t}}} =\sum _{i} \frac{1}{J} \frac{\partial \psi }{\partial {\bar{I}}_{i}}\frac{\partial {\bar{I}}_{i}}{\partial \varvec{F}} \cdot \varvec{F}^{{\textrm{t}}}\\ \end{aligned} \end{aligned}$$

which allows us to separate the individual NINV stress tensor contributions from the ${\partial {\bar{I}}_{i}}/{\partial \varvec{F}}$ terms. We compute all the invariant-specific scalar ${\partial \psi }/{\partial {\bar{I}}_i}$ contributions

19 $$\begin{aligned} \frac{\partial \psi }{\partial {\bar{I}}_{i}} = \sum _{k}^{n_i}\frac{\partial \psi }{\partial {\bar{I}}_{i,k}} = \sum _{k}^{n_i} w_{2, \textrm{k}} \frac{\partial f_{2,k}}{\partial (\circ )} \frac{\partial f_{1,k}}{\partial (\circ )} \frac{\partial f_{0,k}}{\partial {\bar{I}}_{i,k}} \end{aligned}$$

in terms of the first derivatives of our activation functions

20 $$\begin{aligned} \frac{\partial f_{0}}{\partial (\circ )}& = {} \left\{ \begin{array}{c} 1\\ \frac{\frac{|\circ |}{\circ } + 1}{2}\\ \frac{|\circ |}{\circ }\\ \vdots \end{array} \right. \frac{\partial f_{1}}{\partial (\circ )} = \left\{ \begin{array}{c} {1 (\circ )^0}\\ {2 (\circ )^1}\\ {3 (\circ )^2}\\ \vdots \\ m (\circ )^{m-1} \end{array} \right. \nonumber \\ \!\! \frac{\partial f_{2}}{\partial (\circ )} {}&=  {} \left\{ \begin{array}{c} {w_1}\\ {w_1 {\textrm{exp}}(w_1 (\circ ))}\\ {w_1 /(1\text{-}w_1 (\circ ))} \\ \vdots \end{array} \right. \end{aligned}$$

**Tangent stiffness tensor update**

Given that the tangent stiffness tensor ${\mathbb {C}}$ expresses the change of the Cauchy stress tensor $\varvec{\sigma }$ with respect to a change in deformation, its computation requires the second derivatives of the free energy function with respect to the invariants ${\partial ^2 \psi }/{\partial {\bar{I}}_{i,k} \partial {\bar{I}}_{j,k}}$ Here, given the nested structure of our universal material model subroutine, we have ${\partial ^2 \psi }/{\partial {\bar{I}}_{i,k} \partial {\bar{I}}_{j,k}} = 0$, when $i \ne j$. As such, we only have non-zero values

21 $$\begin{aligned} \begin{aligned} \frac{\partial ^2 \psi }{\partial {\bar{I}}_{i,k}^2}=\sum _{k=1}^{n_i} w_{2,k}&\left[ \left( \frac{\partial ^2 f_{2,k}}{\partial (\circ )^2}\left[ \frac{\partial f_{1,k}}{\partial (\circ )}\right] ^2+\frac{\partial f_{2,k}}{\partial (\circ )} \frac{\partial ^2 f_{1,k}}{\partial (\circ )^2}\right) \right. \\&\left. \left[ \frac{\partial f_{0,k}}{\partial {\bar{I}}_{i,k}}\right] ^2+\frac{\partial f_{2,k}}{\partial (\circ )} \frac{\partial f_{1,k}}{\partial (\circ )} \frac{\partial ^2 f_{0,k}}{\partial {\bar{I}}_{i,k}^{2}} \right] \end{aligned} \end{aligned}$$

in terms of the second derivatives of our activation functions,

22 $$\begin{aligned} \displaystyle {\frac{\partial ^2 f_1}{\partial \left( \circ \right) ^2}}&= {} \left\{ \begin{array}{c} 0\\ 2\\ 6\\ \vdots \\ (m^2 -m) (\circ )^{m-2} \end{array} \right. \displaystyle {\frac{\partial ^2 f_2}{\partial \left( \circ \right) ^2}}  {}&= {} \left\{ \begin{array}{c} 0\\ {{w_1}^2{\textrm{exp}}(w_1(\circ ))}\\ {{w_1}^2/{(1-w1(\circ ))^2}} \\ \vdots \end{array} \right. \end{aligned}$$

where the second derivative of the zeroth layer functions, $\partial ^2 f_0/\partial (\circ )^2$, vanishes identically for all three terms.

### Constitutive parameter table

Providing a user interface to employ our developed universal material model subroutine, we design a *constitutive parameter table* that defines the to-be-evaluated constitutive model and parameters during the simulation. Each row of this table represents a neuron of the final layer in our modular constitutive neural network and consists of seven terms: an integer kfinv that defines the index of the invariant ${\bar{I}}_i$ according to the invariant numbering scheme in Eq. (15); three integers kf0, kf1, and kf2 that define the indices of the zeroth, first, and second layer activation functions; and three floats w0, w1, and w2 that define the weights of the zeroth, first, and second layers:

$$\begin{aligned} \begin{array}{l} {\texttt {*PARAMETER \,TABLE, TYPE={\text{"}}UNIVERSAL\_TAB{\text{"}}}} \\ {\texttt {kfinv}_{\texttt {1}},\texttt {kf}_{\texttt {0,1}},\texttt {kf}_{\texttt {1,1}},\texttt {kf}_{\texttt {2,1}},\texttt {w}_{\texttt {0,1}},\texttt {w}_{\texttt {1,1}},\texttt {w}_{\texttt {2,1}}} \\ {\texttt {kfinv}_{\texttt {2}},\texttt {kf}_{\texttt {0,2}},\texttt {kf}_{\texttt {1,2}},\texttt {kf}_{\texttt {2,2}},\texttt {w}_{\texttt {0,2}},\texttt {w}_{\texttt {1,2}},\texttt {w}_{\texttt {2,2}}} \\ {\texttt {kfinv}_{\texttt {3}},\texttt {kf}_{\texttt {0,3}},\texttt {kf}_{\texttt {1,3}},\texttt {kf}_{\texttt {2,3}},\texttt {w}_{\texttt {0,3}},\texttt {w}_{\texttt {1,3}},\texttt {w}_{\texttt {2,3}}} \\ {{{\vdots }}} \end{array}  \end{aligned}$$

The first index of each row selects between the invariants, the second index applies the identity, Macauley brackets, or absolute values to the invariants, $(\circ )$, $\langle \circ \rangle$, $|\circ |$, the third index raises them to the first, second, third, or any higher order powers, $(\circ )^1, (\circ )^2, (\circ )^3, \ldots (\circ )^m$ and the fourth index applies the identity, exponential, or natural logarithm, $(\circ )$, $(\textrm{exp}(\circ )-1)$, $(-\textrm{ln}(1-(\circ )))$, or any other thermodynamically admissible function to these powers. For brevity, we can simply exclude terms with zero weights from the list. We provide further details on the integration and interface of these constitutive parameter tables with an exemplary non-linear FEA solver in Appendix A and B.

### Special cases

To showcase the flexibility and modularity of our universal material model subroutine, we demonstrate how our approach naturally integrates the popular neo Hooke [2], Mooney Rivlin [3, 4], Yeoh [6], polynomial [34], Holzapfel [35], Kaliske [36], and dispersed Holzapfel [37] models into an FEA solver. For each model, we provide the free energy function and its translation into the UNIVERSAL_TAB parameter table for the FEA input file.

*Neo Hooke model*. The free energy function of the compressible linear first invariant neo Hooke model [2]

23 $$\begin{aligned} \psi =C_{10}\left( {\bar{I}}_1-3\right) +\frac{1}{D_1}\left( I_3-1\right) ^2 \end{aligned}$$

translates into the following two-line parameter table

$$\begin{aligned} \begin{array}{l} {{{\texttt {*PARAMETER\, TABLE, TYPE={\text{"}}UNIVERSAL\_TAB{\text{"}}}}}} \\ \begin{array}{ccccccc} 1,&{}1,&{}1,&{}1,&{}1.0,&{}1.0,&{} C_{10} \\ 3,&{}1,&{}2,&{}1,&{}1.0,&{}1.0,&{} 1/D_{1} \end{array} \end{array}  \end{aligned}$$

*Mooney Rivlin model*. The free energy function of the compressible linear first and second invariant Mooney Rivlin model [3, 4]

24 $$\begin{aligned} \psi = C_{10}\left( {\bar{I}}_1-3\right) +C_{01}\left( {\bar{I}}_2-3\right) +\frac{1}{D_1}\left( I_3-1\right) ^2 \end{aligned}$$

translates into the following three-line parameter table

$$\begin{aligned} \begin{array}{l} {{{\texttt {*PARAMETER\, TABLE, TYPE={\text{"}}UNIVERSAL\_TAB{\text{"}}}}}} \\ \begin{array}{ccccccc} 1,&{}1,&{}1,&{}1,&{}1.0,&{}1.0,&{} C_{10} \\ 2,&{}1,&{}1,&{}1,&{}1.0,&{}1.0,&{} C_{01} \\ 3,&{}1,&{}2,&{}1,&{}1.0,&{}1.0,&{} 1/D_{1} \end{array} \end{array}  \end{aligned}$$

*Yeoh model*. The free energy function of the compressible first invariant Yeoh model [6]

25 $$\begin{aligned} \begin{aligned} \psi&= C_{10}\left( {\bar{I}}_1-3\right) +C_{20}\left( {\bar{I}}_1-3\right) ^2+C_{30}\left( {\bar{I}}_1-3\right) ^3 \\&+\frac{1}{D_1}\left( I_3-1\right) ^2+\frac{1}{D_2}\left( I_3-1\right) ^4+\frac{1}{D_3}\left( I_3-1\right) ^6 \end{aligned} \end{aligned}$$

translates into the following six-line parameter table

$$\begin{aligned} \begin{array}{l} {{{\texttt {*PARAMETER \,TABLE, TYPE={\text{"}}UNIVERSAL\_TAB{\text{"}}}}}} \\ \begin{array}{ccccccc} 1,&{}1,&{}1,&{}1,&{}1.0,&{}1.0,&{} C_{10} \\ 1,&{}1,&{}2,&{}1,&{}1.0,&{}1.0,&{} C_{20} \\ 1,&{}1,&{}3,&{}1,&{}1.0,&{}1.0,&{} C_{30} \\ 3,&{}1,&{}2,&{}1,&{}1.0,&{}1.0,&{} 1/D_{1} \\ 3,&{}1,&{}4,&{}1,&{}1.0,&{}1.0,&{} 1/D_{2} \\ 3,&{}1,&{}6,&{}1,&{}1.0,&{}1.0,&{} 1/D_{3} \\ \end{array} \end{array}  \end{aligned}$$

*Polynomial model*. The free energy function of the compressible first invariant polynomial model [34]

26 $$\begin{aligned} \psi =\sum _{i=1}^N C_{i 0}\left( {\bar{I}}_1-3\right) ^i+\sum _{i=1}^N \frac{1}{D_i}\left( I_3-1\right) ^{2 i} \end{aligned}$$

translates into the following parameter table

$$\begin{aligned} \begin{array}{l} {{{\texttt {*PARAMETER \,TABLE, TYPE={\text{"}}UNIVERSAL\_TAB{\text{"}}}}}} \\ \begin{array}{ccccccc} 1,&{}1,&{}1,&{}1,&{}1.0,&{}1.0,&{} C_{10} \\ 1,&{}1,&{}2,&{}1,&{}1.0,&{}1.0,&{} C_{20} \\ 1,&{}1,&{}3,&{}1,&{}1.0,&{}1.0,&{} C_{30} \\ \vdots &{}\vdots &{}\vdots &{}\vdots &{}\vdots &{}\vdots &{} \vdots \\ 1,&{}1,&{}N,&{}1,&{}1.0,&{}1.0,&{} C_{N0} \\ 3,&{}1,&{}2,&{}1,&{}1.0,&{}1.0,&{} 1/D_{1} \\ 3,&{}1,&{}4,&{}1,&{}1.0,&{}1.0,&{} 1/D_{2} \\ 3,&{}1,&{}6,&{}1,&{}1.0,&{}1.0,&{} 1/D_{3} \\ \vdots &{}\vdots &{}\vdots &{}\vdots &{}\vdots &{}\vdots &{} \vdots \\ 3,&{}1,&{}N*2,&{}1,&{}1.0,&{}1.0,&{} 1/D_{N} \\ \end{array} \end{array}  \end{aligned}$$

*Holzapfel model*. The free energy function of the compressible two-fiber family Holzapfel model [35]

27 $$\begin{aligned} \psi & ={} C_{10} \left( {\bar{I}}_1 - 3 \right) + \frac{1}{D} \left( \frac{I_3^2 - 1}{2} - \ln {I_3} \right) \nonumber \\{} & {} + \frac{k_1}{2k_2} \left( \exp {\left[ k_2 \langle {\bar{I}}_{4(11)}-1 \rangle ^2\right] } - 1 \right) \nonumber \\{} & {} + \frac{k_1}{2k_2} \left( \exp {\left[ k_2 \langle {\bar{I}}_{4(22)}-1 \rangle ^2\right] } - 1 \right) \end{aligned}$$

translates into the following six-line parameter table

$$\begin{aligned} \begin{array}{l} {{{\texttt {*PARAMETER \,TABLE, TYPE={\text{"}}UNIVERSAL\_TAB{\text{"}}}}}} \\ \begin{array}{ccccccc} 1,&{}1,&{}1,&{}1,&{}1.0,&{}1.0,&{} C_{10} \\ 4,&{}2,&{}2,&{}2,&{}1.0,&{}k_2,&{} k_1/2k_2 \\ 8,&{}2,&{}2,&{}2,&{}1.0,&{}k_2,&{} k_1/2k_2 \\ 3,&{}1,&{}1,&{}1,&{}1.0,&{}1.0,&{}1/D \\ 3,&{}1,&{}2,&{}1,&{}1.0,&{}0.5,&{}1/D \\ 3,&{}1,&{}1,&{}3,&{}1.0,&{}\text {-}1.0,&{}1/D \end{array} \end{array}  \end{aligned}$$

We provide more details on the modified Ogden volumetric free energy contribution [5] and its derivation into the constitutive parameter table in Eqs. (43) and (44) in Appendix C.

*Kaliske model*. The free energy function of the compressible two-fiber family Kaliske model [36]

28 $$\begin{aligned} \psi= & {} \sum _{i=1}^3 a_i\left( {\bar{I}}_1-3\right) ^i+\sum _{j=1}^3 b_j\left( {\bar{I}}_2-3\right) ^j+\sum _{k=2}^6 c_k\left( {\bar{I}}_{4(11)}-1\right) ^k \nonumber \\{} & {} +\sum _{l=2}^6 d_l\left( {\bar{I}}_{5(11)}-1\right) ^l +\sum _{m=2}^6 e_m\left( {\bar{I}}_{4(22)}-1\right) ^m \nonumber \\{} & {} +\sum _{n=2}^6 f_n\left( {\bar{I}}_{5(22)}-1\right) ^n +\frac{1}{D}\left( \frac{\left( I_3\right) ^2-1}{2}-\ln (I_3)\right) \end{aligned}$$

translates into the following parameter table

$$\begin{aligned} \begin{array}{l} {{{\texttt {*PARAMETER \,TABLE, TYPE={\text{"}}UNIVERSAL\_TAB{\text{"}}}}}} \\ \begin{array}{ccccccccc} 1,&{}1,&{}i,&{}1,&{}1.0,&{}1.0,&{} a_i &{}\, &{}\ldots \\ 2,&{}1,&{}j,&{}1,&{}1.0,&{}1.0,&{} b_j &{}\, &{}\ldots \\ 4,&{}1,&{}k,&{}1,&{}1.0,&{}1.0,&{} c_k &{}\, &{}\ldots \\ 5,&{}1,&{}l,&{}1,&{}1.0,&{}1.0,&{} d_l &{}\, &{}\ldots \\ 8,&{}1,&{}m,&{}1,&{}1.0,&{}1.0,&{} e_m &{}\, &{}\ldots \\ 9,&{}1,&{}n,&{}1,&{}1.0,&{}1.0,&{} f_n &{}\, &{}\ldots \\ 3,&{}1,&{}1,&{}1,&{}1.0,&{}1.0,&{}1/D &{}&{} \\ 3,&{}1,&{}2,&{}1,&{}1.0,&{}0.5,&{}1/D &{}&{} \\ 3,&{}1,&{}1,&{}3,&{}1.0,&{}\text {-}1.0,&{}1/D &{}&{} \end{array} \end{array}  \end{aligned}$$

*Holzapfel dispersion model*. The free energy function of the Holzapfel dispersion model [37]

29 $$\begin{aligned} \begin{aligned} \psi&= C_{10} \left( {\bar{I}}_1 - 3 \right) + \frac{1}{D} \left( \frac{I_3^2 - 1}{2} - \ln {I_3} \right) \\&\quad + \frac{k_1}{2k_2} \left( \exp {\left[ k_2 \langle {\bar{I}}_{1/4(11)}^*-1 \rangle ^2\right] } - 1 \right) \\&\quad + \frac{k_1}{2k_2} \left( \exp {\left[ k_2 \langle {\bar{I}}_{1/4(22)}^*-1 \rangle ^2\right] } - 1 \right) \end{aligned} \end{aligned}$$

uses the two mixed invariants

30 $$\begin{aligned} \begin{aligned} {\bar{I}}_{1/4(11)}^*&= \kappa ({\bar{I}}_1-3) + \left( 1 - 3\kappa \right) ({\bar{I}}_{4(11)}-1)\\ {\bar{I}}_{1/4(22)}^*&= \kappa ({\bar{I}}_1-3) + \left( 1 - 3\kappa \right) ({\bar{I}}_{4(22)}-1) \end{aligned} \end{aligned}$$

where $\kappa$ describes the dispersion of the collagen fibers ranging from $\kappa = 0.0$ for ideally aligned fibers to $\kappa = 1/3$ for isotropically distributed fibers. Using an additional constitutive parameter table definition "MIXED_INV" (see Appendix D), we generalize our universal material model subroutine to include mixed invariants and translate this free energy in the following parameter tables

$$\begin{aligned} \begin{array}{l} {\texttt {*PARAMETER \,TABLE, TYPE={\text{"}}MIXED\_INV{\text{"}}}}\\ {\texttt {1},{\kappa} ,\texttt {0.0,0.0},(1-3\kappa ),\texttt {0.0,0.0,0.0,0.0,0.0,}} \\  {{{\texttt {0.0,0.0,0.0,0.0,0.0,0.0}}}} \\ {{\texttt {2},\kappa, \texttt {0.0,0.0,0.0,0.0,0.0,0.0},(1-3\kappa ),\texttt {0.0,}}} \\  {{{\texttt {0.0,0.0,0.0,0.0,0.0,0.0}}}} \\ {\texttt {*PARAMETER \,TABLE, TYPE={\text{"}}UNIVERSAL\_TAB{\text{"}}}} \\ \begin{array}{ccccccc} 1,&{}1,&{}1,&{}1,&{}1.0,&{}1.0,&{} C_{10} \\ 101,&{}2,&{}2,&{}2,&{}1.0,&{}k_2,&{} k_1/2k_2 \\ 102,&{}2,&{}2,&{}2,&{}1.0,&{}k_2,&{} k_1/2k_2 \\ 3,&{}1,&{}1,&{}1,&{}1.0,&{}1.0,&{}1/D \\ 3,&{}1,&{}2,&{}1,&{}1.0,&{}0.5,&{}1/D \\ 3,&{}1,&{}1,&{}3,&{}1.0,&{}\text {-}1.0,&{}1/D \end{array} \end{array}  \end{aligned}$$

## Illustrative applications

In the following sections, we showcase examples of soft matter systems where our universal material model subroutine naturally integrates both existing and newly discovered constitutive models from the material point level to the structural level.

### The human brain

**Fig. 3:**
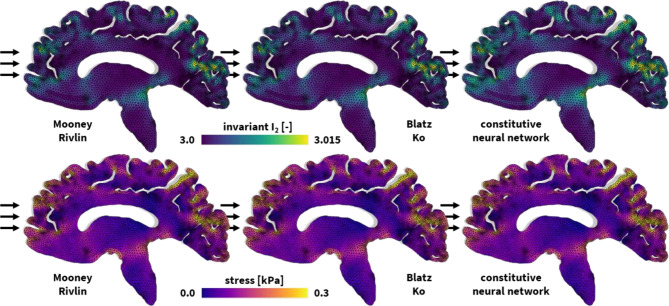
**Universal material modeling of the human brain**. Deformation and stress profiles for frontal impact to the human brain. The finite element models simulate the deformation and internal tissue loading corresponding to best-fit Mooney Rivlin, Blatz Ko, and newly discovered constitutive models from left to right. All simulations leverage our universal material model subroutine and only differ in the definition of the UNIVERSAL_TAB constitutive parameter table in the finite element analysis input file.

Brain tissue is among the softest and most vulnerable tissues in the human body [38]. The tissue’s delicate packing of neurons, glial cells, and extracellular matrix functionally regulates most vital processes in the human body and governs human cognition, learning, and consciousness [39]. As mechanics play a crucial role in neuronal function and dysfunction [40], understanding the mechanical behavior of brain tissue is essential for anticipating how the brain will respond to injury, how it evolves during its development, or how it remodels as disease advances. Computational models play a crucial role in this endeavor, allowing researchers to simulate the multi-faceted behavior of brain tissue and explore the biomechanical role of mechanical forces in health and disease [41–44]. These models require adequate constitutive models that capture the complex and unique characteristics of this ultrasoft, highly adaptive, and heterogeneous tissue.

**Constitutive modeling**

Over the past decade, various research groups around the world have made significant process in the experimental and constitutive characterization of human brain tissue [45]. This has led to multiple competing constitutive models to characterize the behavior of gray and white matter tissue. Most notably, neo Hooke [2], Blatz Ko [46], Mooney Rivlin [3, 4], Demiray [47], Gent [48], and Holzapfel [35] models were proposed as successful candidates to characterize the stress-stretch response of these tissues. Given brain tissue’s intricate behavior, fitting a constitutive model to one single loading mode, tension, compression, or shear, does not generalize well to the other modes [25, 49]. Therefore, we consider a widely-used benchmark dataset where $5 \times 5 \times 5\,\hbox {mm}^3$ human brain samples were tested in tension, compression, and shear [38, 45, 50]. We concomitantly discover and fit the best possible constitutive models considering these loading modes together and find the following three best models and parameters [25].

The Mooney Rivlin model [3, 4]

31 $$\begin{aligned} \psi =\frac{1}{2}\mu _1\left( {\bar{I}}_1-3\right) +\frac{1}{2}\mu _2\left( {\bar{I}}_2-3\right) \end{aligned}$$

with parameters $\mu _1=0.0021$ kPa, $\mu _2=1.8817$ kPa for the gray matter cortex, and $\mu _1=0.0168$ kPa, $\mu _2=0.9697$ kPa for the white matter corona radiata. This translates into

$$\begin{aligned} \begin{array}{l} {{{\texttt {*PARAMETER\, TABLE, TYPE={\text{"}}UNIVERSAL\_TAB{\text{"}}}}}} \\ \begin{array}{ccccccc} 1,&{}1,&{}1,&{}1,&{}1.0,&{}1.0,&{} \mu _1/2 \\ 2,&{}1,&{}1,&{}1,&{}1.0,&{}1.0,&{} \mu _2/2 \end{array} \end{array}  \end{aligned}$$

The Blatz Ko model [46]

32 $$\begin{aligned} \psi =\frac{1}{2}\mu \left( {\bar{I}}_2-3\right) \end{aligned}$$

with parameters $\mu =1.9043$ kPa for the gray matter cortex, and $\mu =0.9556$ kPa for the white matter corona radiata. This translates into

$$\begin{aligned} \begin{array}{l} {{{\texttt {*PARAMETER\, TABLE, TYPE={\text{"}}UNIVERSAL\_TAB{\text{"}}}}}} \\ {{{\texttt {2,1,1,1,1.0,1.0,} \,\mu \texttt {/2 }}}} \\ \end{array}  \end{aligned}$$

Our newly discovered six-term model [25, 26]

33 $$\begin{aligned} \begin{aligned} \psi&= \mu _1 \left[ {\bar{I}}_2-3\right] +\frac{a_1}{2b_1}\left[ \exp \left( b_1\left[ {\bar{I}}_2-3\right] \right) -1\right] \\&\quad -\frac{\alpha _1}{2\beta _1} \ln \left( 1-\beta _1\left[ {\bar{I}}_2-3\right] \right) +\mu _2\left[ {\bar{I}}_2-3\right] ^2 \\&\quad +\frac{a_2}{2b_2}\left[ \exp \left( b_2\left[ {\bar{I}}_2-3\right] ^2\right) -1\right] \\ {}&\quad -\frac{\alpha _2}{2\beta _2} \ln \left( 1-\beta _2\left[ {\bar{I}}_2-3\right] ^2\right) \end{aligned} \end{aligned}$$

with non-zero terms $\alpha _1=1.2520$ kPa, $\beta _1=0.9875$, $\mu _2=3.8007$ kPa, $a_2=6.2285$ kPa, $b_2=1.6495$, $\alpha _2=4.6743$ kPa, and $\beta _2=1.6663$ for the gray matter cortex and $\mu _1=0.2215$ kPa, $a_1=0.2350$ kPa, $b_1=0.2398$, $a_2=6.3703$ kPa, $b_2=1.8893$, $\alpha _2=4.5065$ kPa, and $\beta _2=1.1789$ for the white matter corona radiata. We translate this model into the following six-line parameter table of our universal material model:

$$\begin{aligned} \begin{array}{l} {{{\texttt {*PARAMETER\, TABLE, TYPE={\text{"}}UNIVERSAL\_TAB{\text{"}}}}}} \\ \begin{array}{ccccccc} 2,&{}1,&{}1,&{}1,&{}1.0,&{}1.0,&{} \mu _1 \\ 2,&{}1,&{}1,&{}2,&{}1.0,&{}b_1,&{} a_1/2b_1 \\ 2,&{}1,&{}1,&{}3,&{}1.0,&{}\beta _1,&{} \alpha _1/2\beta _1 \\ 2,&{}1,&{}2,&{}1,&{}1.0,&{}1.0,&{} \mu _2 \\ 2,&{}1,&{}2,&{}2,&{}1.0,&{}b_2,&{} a_2/2b_2 \\ 2,&{}1,&{}2,&{}3,&{}1.0,&{}\beta _2,&{} \alpha _2/2\beta _2 \\ \end{array} \end{array}  \end{aligned}$$

The Mooney Rivlin, the Blatz Ko, and the newly discovered six-term material models have a gray and white matter goodness of fit of $\textrm{R}^2 = 0.8784$ and $\textrm{R}^2 = 0.7414$, $\textrm{R}^2 = 0.8809$ and $\textrm{R}^2 = 0.7355$, and $\textrm{R}^2 = 0.9306$ and $\textrm{R}^2 = 0.8361$ respectively to the combined tension, compression, and shear testing data [25].

**Simulation**

Utilizing our universal material model subroutine, we incorporate these brain models into a realistic vertical head impact finite element simulation [26]. Based on magnetic resonance images [51], we create the two-dimensional sagittal finite element model in Fig. 3. In this model, gray and white matter are spatially discretized using 6,182 gray and 5,701 white linear triangular elements, resulting in 6,441 nodes, and 12,882 degrees of freedom in total. We embed our model into the skull using spring support at the free boundaries and apply a frontal impact to the brain that we represent with all three models, the Mooney Rivlin, Blatz Ko, and new discovered models, as shown in Fig. 3. While our results showcase equal spatial stress magnitudes across the brain for all three, the Mooney Rivlin, Blatz Ko, and constitutive neural network models, the simulation underestimates the maximum deformation for the Mooney Rivlin and Blatz Ko models compared to the constitutive neural network model.

### Skin

Skin is the largest organ of the human body [52]. It serves vital functions for our survival such as being the first line of defense against mechanical injury while at the same time allowing us to move and interact with the world [53]. Surgery of any kind entails skin rupture and manipulation [54]. Especially during reconstructive procedures, skin tissues are subjected to extreme deformations [55]. The complex stress field generated by skin tissue manipulation has a direct effect on the subsequent wound healing response, with excessive stress causing increased inflammatory response that can lead to fibrosis [56]. In some cases, excessive stress can even result in tissue necrosis [57]. Thus, accurate computational models of skin are key to design safe reconstructive surgical procedures.

**Fig. 4:**
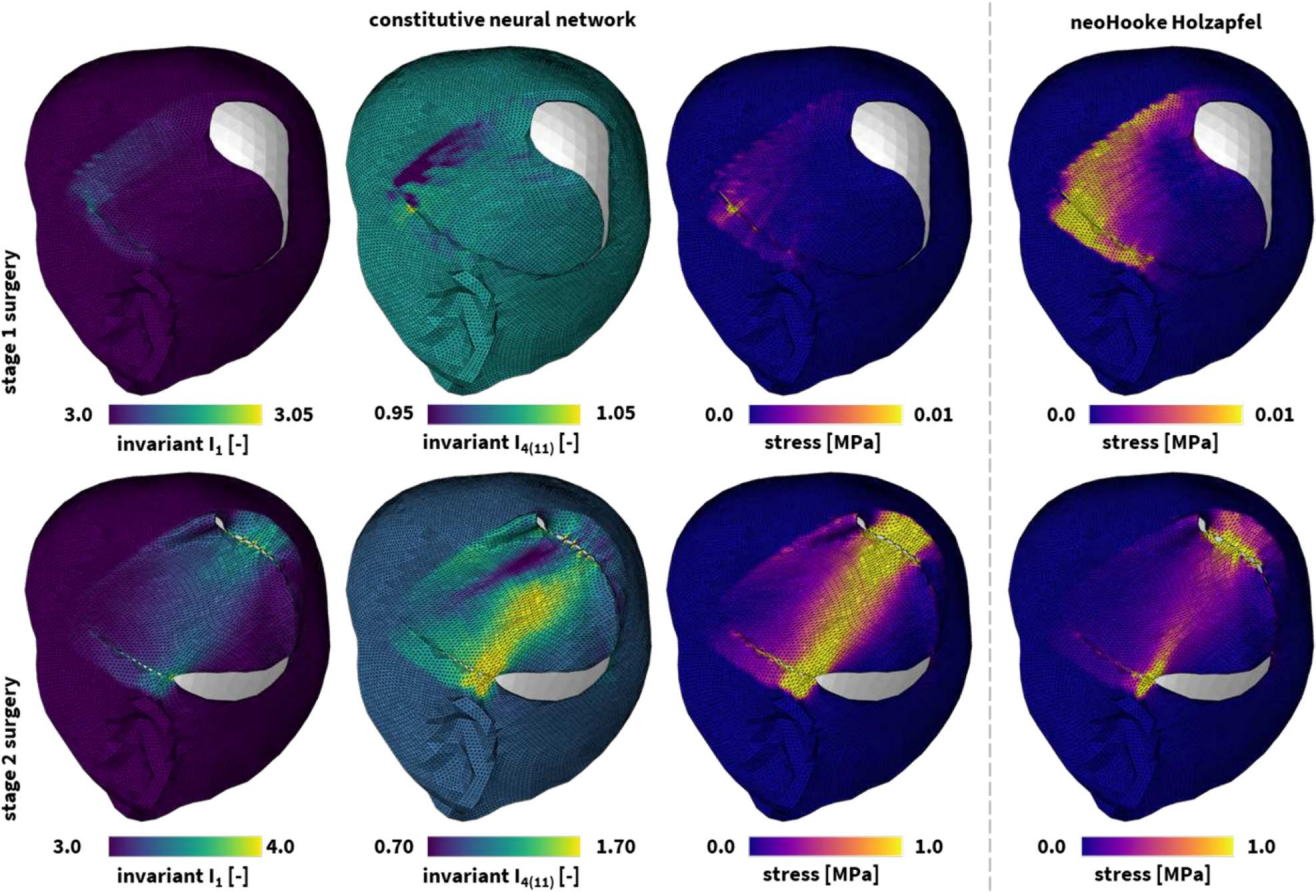
**Universal material modeling of skin**. Deformation and stress profiles in the human scalp following a melanoma resection reconstruction procedure. The finite element models simulate the deformation and internal tissue loading corresponding a two-stage flap rotation and suturing procedure, with the first stage shown in the top row and the second stage shown in the bottom row. The remaining wound is closed with a skin graft to avoid excessive tissue stresses and damage. Both tissue manipulations are modeled using the best-fit constitutive neural network model in the three left columns. For comparison, we also showcase the resulting stress profiles for the best-fit neo Hooke Holzapfel model in the right column. All simulations leverage our universal material model subroutine and only differ in the definition of the UNIVERSAL_TAB constitutive parameter table in the finite element analysis input file

**Constitutive modeling**

Skin modeling has received significant attention for more than half a century [58, 59]. Isotropic models such as the neo Hooke [2] or Mooney Rivlin [3, 4] models have been used, but show significant limitations. Not only do they fail to describe the anisotropy of skin, they also lack the ability to capture this tissue’s rapid strain-stiffening behavior [58]. To overcome these issues, we examine combined uniaxial and biaxial tensile testing data of porcine skin tissue samples [60, 61] to discover more accurate material models that depict the anisotropic stress-stretch behavior. First, we fit the microstructure-inspired Holzapfel model [35],

34 $$\begin{aligned} \psi =\frac{1}{2} \mu \left[ {\bar{I}}_1-3\right] +\frac{1}{2} \frac{a_4}{b_4}\left[ \exp \left( b_4\langle {\bar{I}}_{4(11)}-1\rangle ^2\right) -1\right] . \end{aligned}$$

This model was originally developed for arterial tissues and combines the isotropic linear first invariant neo Hooke term, $\left[ {\bar{I}}_1-3\right]$, with an anisotropic quadratic exponential fourth invariant term, $\langle {\bar{I}}_{4(11)}-1\rangle$, along the collagen fiber direction. Here, our best possible fit to the combined uniaxial and biaxial testing data results in $\mu = 0.2492$ MPa, $a_4 = 0.1054$ MPa, and $b_4 = 10.7914$. We naturally incorporate this constitutive model and parameters in our universal material model subroutine using the following two-line parameter table

$$\begin{aligned} \begin{array}{l} {{{\texttt {*PARAMETER \,TABLE, TYPE={\text{"}}UNIVERSAL\_TAB{\text{"}}}}}} \\ \begin{array}{ccccccc} 1,&{}1,&{}1,&{}1,&{}1.0,&{}1.0,&{} \mu \\ 4,&{}2,&{}2,&{}2,&{}1.0,&{}b_4,&{} a_4/2b_4 \end{array} \end{array}  \end{aligned}$$

To address the poor goodness of fit $\textrm{R}^2 = 0.6857$ of the neo Hooke Holzapfel model, we adopt a tranversely isotropic constitutitive neural network to discover a more accurate model [27]. From a library of $2^{16}=65,536$ possible combinations of terms, we discover a model in two exponential quadratic terms,

35 $$\begin{aligned} \psi=\, & {} \frac{a_1}{2b_1} ( \exp {[b_1 ({\bar{I}}_{1}-3 )^2 ]} - 1 ) \nonumber \\ {}{} & {} + \frac{a_4}{2b_4} ( \exp {[b_4 \langle {\bar{I}}_{4(11)}-1 \rangle ^2 ]} - 1 ) \end{aligned}$$

with parameters $a_1 = 1.3291$ MPa, $b_1 =0.8207$, $a_4= 0.2656$ MPa, and $b_4= 0.3921$ [27]. To integrate this new model into a finite element simulation, we incorporate the following two parameter lines in our universal material subroutine

$$\begin{aligned} \begin{array}{l} {{{\texttt {*PARAMETER \,TABLE, TYPE={\text{"}}UNIVERSAL\_TAB{\text{"}}}}}} \\ \begin{array}{ccccccc} 1,&{}1,&{}2,&{}2,&{}1.0,&{}b_1,&{} a_1/2b_1 \\ 4,&{}2,&{}2,&{}2,&{}1.0,&{}b_4,&{} a_4/2b_4 \end{array} \end{array}  \end{aligned}$$

In contrast to the neo Hooke Holzapfel model with a goodness of $\textrm{R}^2 = 0.6857$, our newly discovered model has a mean good of fit $\textrm{R}^2 = 0.8629$ for the biaxial skin testing data [27].

**Simulation**

Leveraging our universal material subroutine, we integrate both material models in a finite element simulation of a 62-year-old adult male patient undergoing reconstructive surgery following surgical melanoma resection [55]. A three-dimensional patient specific geometry was obtained via multi-view stereo reconstruction of a sequence of photos taken in the operating room before and after surgery. The scalp was approximated based on the skin surface and spatially discretized using 75,282 linear tetrahedral elements and 25,394 nodes, leading to a total 76,182 degrees of freedom. Our simulation recapitulates the closure of the resected tissue defect by imposing nodal constraints to nodes on either edge of the defect to mimic sutures used to close the wound. Figure 4 showcases the deformation and internal tissue tension profiles following the two-step surgical skin reconstruction procedure. We clearly observe the limited tissue deformation and loading profiles during the first stage in the top row. In contrast, during the second stage surgery in the bottom row, substantial deformations develop across the skin. Specifically, we appreciate the regional differences between the isotropic ${\bar{I}}_1$ and anisotropic ${\bar{I}}_{4(11)}$ deformation invariants. Figure 4 also showcases noticeable stress profile differences between the newly discovered material model and the neo Hooke Holzapfel model in the third and fourth columns. In the lower stretch regimes shown in the first stage reconstruction, the neo Hooke Holzapfel model clearly overestimates the stresses in the skin. In the higher stretch regimes, shown during the second stage reconstruction in the bottom, the neo Hooke Holzapfel fit underestimates the stresses in the tissue. While a modeling-based overestimation of the stress state holds limited risks from a medical point of view, an underestimation could have harmful consequences as clinical decisions co-informed by such models could cause excessive tissue damage and scarring. Figure 4 showcases the crucial aspect that proper constitutive modeling and calibration plays in this regard, in which the neo Hooke Holzapfel model, which does not properly capture skin tissue’s strain-stiffening, underestimates the tissue stress in comparison to the more accurate newly discovered model.

### Human arteries

**Fig. 5:**
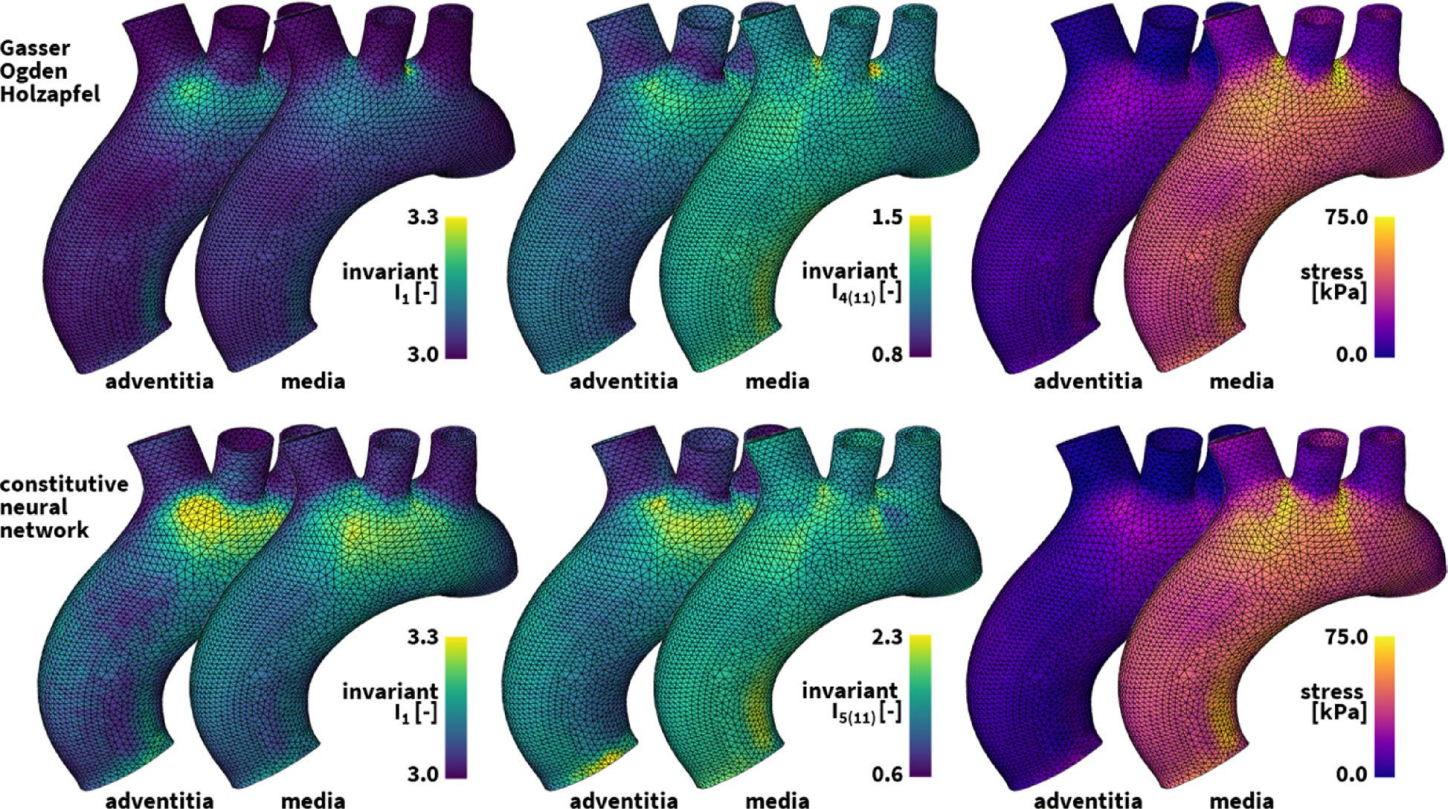
**Universal material modeling of human arteries**. Diastolic deformation and stress profiles in the media and adventitia layer of the human ascending aortic arch. The finite element models simulate the deformation and internal tissue loading corresponding to the best-fit Holzapfel dispersion model in the top row and newly discovered model in the bottom row. Both simulations leverage our universal material model subroutine and only differ in the definition of the UNIVERSAL_TAB constitutive parameter table in the finite element analysis input file

Computational simulations play a pivotal role in understanding and predicting the biomechanical factors of a wide variety of arterial diseases [32, 62–64]. In vascular medicine, knowing the precise stress and strain fields across the vascular wall is critical for understanding the formation, growth, and rupture of aneurysms and dissections [65–67]; for identifying high-risk regions of plaque formation, rupture, and thrombosis [68, 69]; and for optimizing stent design and surgery [70, 71].

**Constitutive modeling**

Over the past four decades, various phenomenological polynomial [72, 73], exponential [74], logarithmic [75], and exponential-polynomial [28, 76, 77] models have been proposed to describe the non-linear elastic, anisotropic, quasi-incompressible behavior of arterial tissue. Recently, microstructurally-informed models were brought forward, including symmetric two- and four-fiber family models [35, 37, 78], either symmetric or unsymmetric [79]. All these material models can fit uniaxial and biaxial arterial tissue testing data, but do not always generalize well to off-axis testing regimes [80].

We consider biaxial tensile testing of thoracic aortic tissue samples at five differing circumferential-axial stretch ratios [81, 82]. Using data-driven constitutive neural networks, we discover the most appropriate arterial material model. From a library of $2^{16}=65,536$ possible combinations of terms, we discover

36 $$\begin{aligned} \psi=\, & {} \frac{\mu _1}{2} [ {\bar{I}}_{1}-3 ] + \frac{a}{2b} ( \exp {[b ({\bar{I}}_{1}-3 ) ]} - 1 ) \nonumber \\{} & {} + \sum _{i=1,2} \frac{1}{2} \mu _5 \langle {\bar{I}}_{5(i i)}-1 \rangle ^2 \end{aligned}$$

with an isotropic linear and exponential linear first invariant term and an anisotropic quadratic fifth invariant term. Our best-fit parameters read $\mu _1 = 33.45$ kPa, $a = 3.74$ kPa, $b = 6.66$, $\mu _5 = 2.17$ kPa for the media at an angle $\alpha = \pm 7.00^{\circ }$, with a goodness of fit of $\textrm{R}^2 = 0.9682$, and $\mu _1 = 8.30$ kPa, $a = 1.42$ kPa, $b = 6.34$, $\mu _5 = 0.49$ kPa for the adventitia at an angle $\alpha = \pm 66.78^{\circ }$, with a goodness of fit of $\textrm{R}^2 = 0.9650$. This translates into the following four-line parameter table of our universal material model,

$$\begin{aligned} \begin{array}{l} {{{\texttt {*PARAMETER\, TABLE, TYPE={\text{"}}UNIVERSAL\_TAB{\text{"}}}}}} \\ \begin{array}{ccccccc} 1,&{}1,&{}1,&{}1,&{}1.0,&{}1.0,&{} \mu _1/2 \\ 1,&{}1,&{}1,&{}2,&{}1.0,&{}b,&{} a/2b \\ 5,&{}2,&{}2,&{}1,&{}1.0,&{}1.0,&{} \mu _5/2 \\ 9,&{}2,&{}2,&{}1,&{}1.0,&{}1.0,&{} \mu _5/2 \\ \end{array} \end{array}  \end{aligned}$$

Alternatively, in the classical microstructure-inspired dispersion type Holzapfel model [68]

37 $$\begin{aligned} \psi =\frac{1}{2} \mu [{\bar{I}}_1-3 ] +\sum _{i=1,2} \frac{a}{2b} ( \exp {[b \langle {\bar{I}}_{1/4(ii)}^*-1 \rangle ^2]} - 1 ) \end{aligned}$$

our best-fit parameters are $\mu = 48.68$ kPa, $a = 6.67$ kPa, $b = 23.17$, $\kappa = 0.074$ for the media at $\alpha = \pm 7.00^{\circ }$, with a goodness of fit of $\textrm{R}^2 = 0.9228$, and $\mu = 13.22$ kPa, $a = 0.93$ kPa, $b = 12.06$, $\kappa = 0.091$ for the adventitia at $\alpha = \pm 66.78^{\circ }$, with a goodness of fit of $\textrm{R}^2 = 0.9525$. We translate this model into the following parameter table of our universal material model

$$\begin{aligned} \begin{array}{l} {\texttt {*PARAMETER\, TABLE, TYPE={\text{"}}MIXED\_INV{\text{"}}}} \\ {\texttt {1},\kappa, \texttt {0.0,0.0},(1-3\kappa ),\texttt {0.0,0.0,0.0,0.0,0.0,}} \\ {{{\texttt {0.0,0.0,0.0,0.0,0.0,0.0}}}} \\ {\texttt {2},\kappa ,\texttt {0.0,0.0,0.0,0.0,0.0,0.0},(1-3\kappa ),\texttt {0.0}}, \\  {{\texttt {0.0,0.0,0.0,0.0,0.0,0.0}}} \\ {\texttt {*PARAMETER\, TABLE, TYPE={\text{"}}UNIVERSAL\_TAB{\text{"}}}} \\ \begin{array}{ccccccc} 1,&{}1,&{}1,&{}1,&{}1.0,&{}1.0,&{} \mu /2 \\ 101,&{}2,&{}2,&{}2,&{}1.0,&{}b,&{} a/2b \\ 102,&{}2,&{}2,&{}2,&{}1.0,&{}b,&{} a/2b \end{array} \end{array}  \end{aligned}$$

**Simulation**

Using our universal material subroutine, we integrate both models in a finite element simulation of the human aortic arch under hemodynamic loading conditions [83]. Our aortic arch geometry is extracted from high-resolution magnetic resonance images of a healthy, 50th percentile U.S. male [84]. We assume an average aortic wall thickness of 3.0 mm, where the inner 75% of the wall make up the media and the outer 25% make up the adventitia. We discretize our geometry using 60,684 linear tetrahedral elements for the media and 30,342 linear tetrahedral elements for the adventitia, leading to a total 61,692 degrees of freedom. The local collagen fiber angles against the circumferential direction are ± 7.00$^{\circ }$ in the media and ± 66.78$^{\circ }$ in the adventitia and are locally defined as a vector field variable for each element. We use continuum distributed coupling boundary conditions at the aortic outlets to constrain the arch in space [85], and leverage Neumann boundary conditions to simulate the hemodynamic loading conditions the aortic arch undergoes during a single cardiac cycle. Figure 5 showcases the computed diastolic stresses in the media and the adventitia for both our newly discovered model and the microstructure-informed dispersion-type Holzapfel model [28]. Comparing the best-fit Holzapfel dispersion material model with a goodness of fit of $\textrm{R}^2 = 0.9228$ for the media and $\textrm{R}^2 = 0.9525$ for the adventitia to the newly discovered model with a goodness of fit of $\textrm{R}^2 = 0.9682$ for the media and $\textrm{R}^2 = 0.9650$ for the adventitia, we observe substantial differences in both the isotropic and anisotropic spatial deformation components as well as the overall aortic arch deformation under the same loading and boundary conditions. Figure 5 also highlights higher stress magnitudes for the best-fit neural network model.

### Heart valves

The tricuspid valve is our right atrioventricular valve which ensures unidirectional blood flow through the right side of the heart. Often as a result of other primary diseases [86, 87], a diseased tricuspid valve can fail to close and regurgitate. Tricuspid valve disease affects over one million Americans and is associated with increased patient mortality and morbidity [88, 89]. Computational models of the tricuspid valve provide valuable insights into the workings of the valve, and have been used to increase our understanding of the progression of valve disease [90] and to work towards improved repair outcomes [91].

**Fig. 6:**
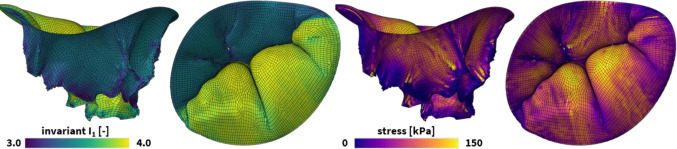
**Universal material modeling of heart valves.** Personalized tricuspid valve loading during the cardiac cycle. The finite element models simulate the deformation, left, and internal tissue loading, right, in response to the inter-ventricular pressure changes from end-diastole to end-systole. The tricuspid valve is shown from a side and top view. Each valvular leaflet leverages our universal material model subroutine and only differs in the definition of the UNIVERSAL_TAB constitutive parameter table in the finite element analysis input file.

**Constitutive modeling**

Numerous studies have investigated the mechanical behavior of atrioventricular valve leaflets. Valvular leaflets exhibit a pronounced anisotropy and a non-linear behavior, motivating an anisotropic exponential material model to capture this complex material behavior [92]. Others have used microstructurally-informed models [37, 93] or anisotropic exponential Fung-type models [94] to capture the material response of the tricuspid valve leaflets. However, the tricuspid valve leaflets specifically only exhibit slight anisotropy [95]. To improve the ease of use in computational models, recent studies have proposed a simplified isotropic Fung-type exponential function [96]. Leveraging force-controlled 400 mN equibiaxial mechanical tests on 7 $\times$ 7 mm valve leaflet tissue samples [97], we fit the following two-term isotropic exponential Fung-Type model [98]

38 $$\begin{aligned} \psi = \frac{c_0}{2} [{\bar{I}}_{1}-3] + \frac{c_1}{2} ( \exp {\left[ c_2 ({\bar{I}}_{1}-3 \right) ^2 ]} - 1 ) \end{aligned}$$

with an isotropic linear first invariant term describing the response at small-strains and under compression and an exponential first invariant term determining the strain-stiffening response under large strains [96]. Our best-fit parameters are $c_0=1.0$ kPa, $c_1=0.124$ kPa, $c_2= 4.57$ for the anterior, $c_0=1.0$ kPa, $c_1=0.188$ kPa, $c_2=14.86$ for the posterior, and $c_0=1.0$ kPa, $c_1=0.191$ kPa, $c_2=17.75$ for the septal leaflets. To incorporate this constitutive model in our universal material subroutine, we define the following two parameter lines

$$\begin{aligned} \begin{array}{l} {{{\texttt {*PARAMETER \,TABLE, TYPE={\text{"}}UNIVERSAL\_TAB{\text{"}}}}}} \\ \begin{array}{ccccccc} 1,&{}1,&{}1,&{}1,&{}1.0,&{}1.0,&{} c_0/2 \\ 1,&{}1,&{}2,&{}2,&{}1.0,&{}c_2,&{} c_1/2 \end{array} \end{array}  \end{aligned}$$

**Simulation**

Using our universal material subroutine, we integrate the constitutive behavior of all three leaflets into a personalized finite element model of the tricuspid valve, the Texas 1.1 TriValve [98, 99]. Through personalized pressure and annular displacement recordings in the realistic hemodynamic environment of an organ preservation system and image-based planimetry meaurements on the excised valve, a three-dimensional reconstruction of the tricuspid valve is build at end-diastole. The valve and chordae geometries are spatially discretized using 8,283 linear quadrilaterial shell elements and 4,169 three-dimensional linear multi-segmented truss elements, resulting in a total 25,761 degrees of freedom. By imposing the recorded personalized annular displacements and an end-systolic transvalvular pressure of 22.95 mmHg on the ventricular surface of the valve, we simulate valvular loading from end-diastole to end-systole. Figure 6 showcases the resulting deformation and maximum principal stress contours in the tricuspid valve. Notably, the varying stiffnesses of the anterior, septal, and posterior leaflets result in noticeable differences in the first invariant of the Cauchy-Green deformation tensor, but in comparable maximum principal stress profiles across the leaflets.

### The human heart

**Fig. 7:**
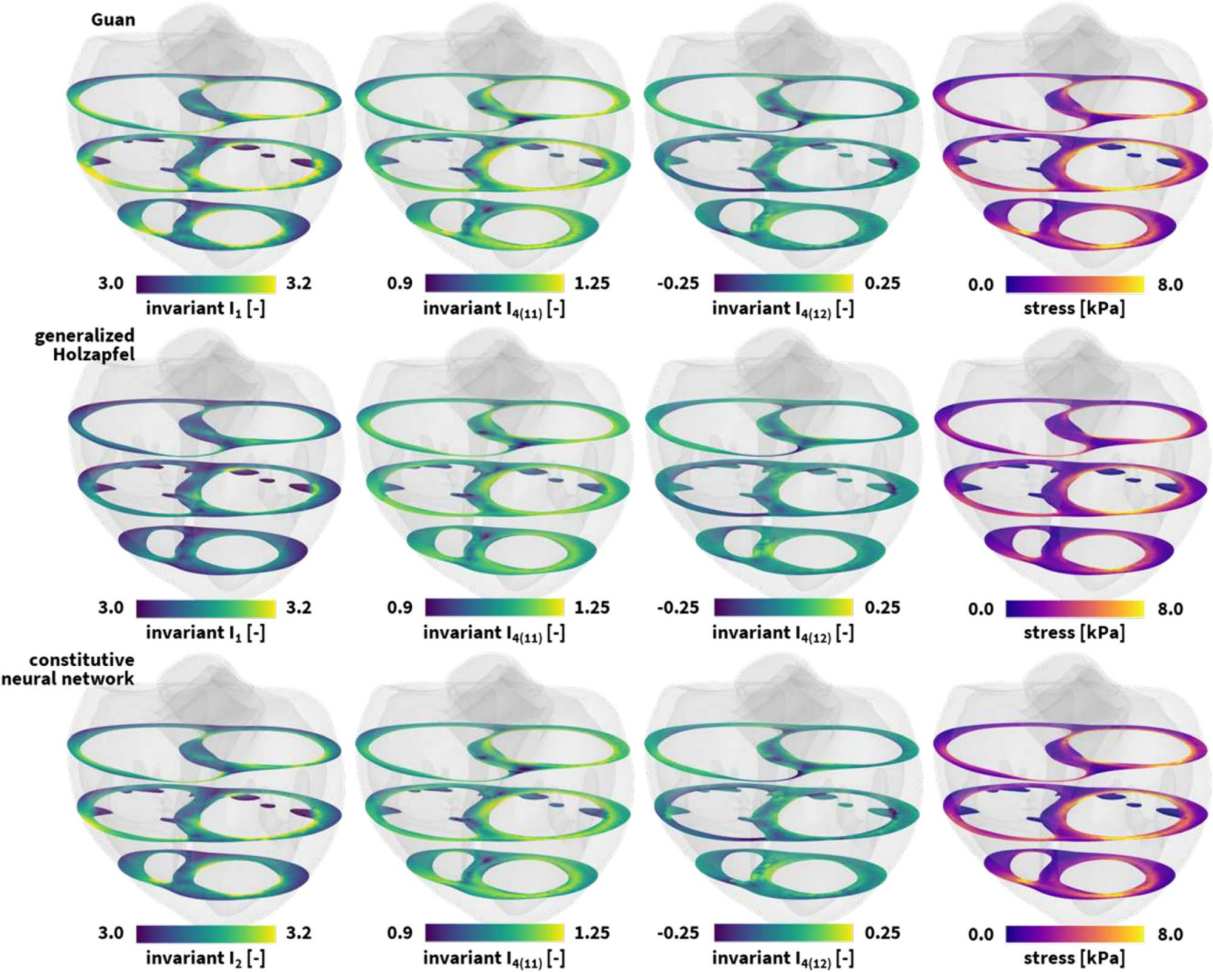
**Universal material modeling of the human heart.** Personalized isotropic and directional deformation invariant and maximum principal stresses stress profiles, in short-axis slices frontal views, resulting from a healthy left and right ventricular end-diastolic pressure loading of 8mmHg and 4mmHg. The finite element models simulate the deformation and internal tissue loading corresponding to the best-fit Guan model in the top row, the generalized Holzapfel model in the middle row, and the newly discovered model in the bottom row. All three simulations leverage our universal material model subroutine and only differ in the definition of the UNIVERSAL_TAB constitutive parameter table in the finite element analysis input file

Cardiac disorders are a leading cause of morbidity and death worldwide [100]. Computational models of cardiac function hold immense potential to contribute to our understanding of health and disease, improve our diagnostic analyses, and optimize personalized intervention [84, 101–105]. For example, corrective surgeries in obstructive cardiomyopathy [106] and congenital heart defects [107], the replacement of diseased valves [108], or the implantation of a cardiac assist device [109] all involve complex and delicate procedures that demand careful planning and simulation to ensure their success. Crucially, the accuracy and reliability of these computational models hinge on precise constitutive modeling of the underlying mechanical behavior of myocardial tissue.

**Constitutive modeling**

Research on constitutive models that accurately describe passive myocardial mechanics spans over five decades. One of the earliest models described cardiac muscle tissue as an isotropic hyperelastic material [110]. Later, with increasing experimental insights, more sophisticated transversely isotropic [111, 112], and eventually orthotropic [19, 113] constitutive models were introduced with three principal directions, the fiber direction $\varvec{f}$ as principal direction $i=1$, the sheet direction $\varvec{s}$ as principal direction $i=2$, and the normal direction $\varvec{n}$ as principal direction $i=3$. This latter orthotropic Holzapfel material model is currently one of the most popular models for heart muscle tissue and fits simple shear tests of myocardial tissue well [114]. Nevertheless, it displays limitations when simultaneously fitted to different loading modes [115]. Therefore, we consider triaxial shear and biaxial extension tests on human myocardial tissue [116], and use these data to discover the best possible model and parameters to characterize both loading conditions combined [29].

We begin with the four-term Guan model [115] that features an exponential linear term in the first invariant ${\bar{I}}_1$, exponential quadratic terms in the fiber and normal fourth invariants ${\bar{I}}_{{{4(11)}}}$ and ${\bar{I}}_{{{4(33)}}}$, and an exponential quadratic term in the fiber-sheet coupling invariant ${\bar{I}}_{{{4(12)}}}$,

39 $$\begin{aligned} \begin{aligned} \psi&= \frac{a}{2b} [ \exp (b[{\bar{I}}_1 - 3 ]) ] +\frac{a_{{\textrm{f}}}}{2b_{{\textrm{f}}}} [ \exp \left( b_{{\textrm{f}}} \langle {\bar{I}}_{{{4(11)}}} -1 \rangle ^2 \right) - 1 ] \\&\quad + \frac{a_{{\textrm{n}}}}{2b_{{\textrm{n}}}} [ \exp (b_{{\textrm{n}}} \langle {\bar{I}}_{{{4(33)}}} -1 \rangle ^2 ) - 1] \\&\quad +\frac{a_{{\textrm{fs}}}}{2b_{{\textrm{fs}}}} [ \exp ( b_{{\textrm{fs}}} [ I_{{{4(12)}}} ]^2 ) - 1]. \end{aligned} \end{aligned}$$

Calibrating this model simultaneously on biaxial tensile and triaxial shear data for human myocardial tissue, we obtain a mean goodness of fit $\textrm{R}^2 = 0.867$ for parameters $a = 0.782$ kPa, $b = 7.248$, $a_{{\textrm{f}}} = 4.488$ kPa, $b_{{\textrm{f}}} = 14.571$, $a_{{\textrm{n}}} = 2.513$ kPa, $b_{{\textrm{n}}} = 10.929$, $a_{{\textrm{fs}}} = 0.436$ kPa, and $b_{{\textrm{fs}}} = 4.959$. To incorporate this constitutive model in our universal material subroutine, we define the following four parameter lines,

$$\begin{aligned} \begin{array}{l} {{{\texttt {*PARAMETER\, TABLE, TYPE={\text{"}}UNIVERSAL\_TAB{\text{"}}}}}} \\ \begin{array}{ccccccc} 1,&{}1,&{}1,&{}2,&{}1.0,&{}b,&{} a/2b \\ 4,&{}2,&{}2,&{}2,&{}1.0,&{}b_f,&{} a_f/2b_f \\ 14,&{}2,&{}2,&{}2,&{}1.0,&{}b_n,&{} a_n/2b_n \\ 6,&{}1,&{}2,&{}2,&{}1.0,&{}b_{fs},&{} a_{fs}/2b_{fs} \end{array} \end{array}  \end{aligned}$$

Next, we consider the seven-term generalized orthotropic Holzapfel model [19] which features an exponential linear term in the first invariant ${\bar{I}}_1$, exponential quadratic terms of all fourth anisotropic invariants ${\bar{I}}_{{{4(11)}}}$, ${\bar{I}}_{{{4(22)}}}$, ${\bar{I}}_{{{4(33)}}}$, and an exponential quadratic term in all fourth coupling invariants ${\bar{I}}_{{{4(12)}}}$, ${\bar{I}}_{{{4(13)}}}$, ${\bar{I}}_{{{4(23)}}}$.

40 $$\begin{aligned} \displaystyle {\psi }=\, & {} \displaystyle {\frac{a}{2b}} \displaystyle {[ \exp (b[{\bar{I}}_1 - 3 ]) ]} + \displaystyle {\frac{a_{{\textrm{f}}}}{2b_{{\textrm{f}}}}} \displaystyle {[ \exp (b_{{\textrm{f}}} \langle {\bar{I}}_{{{4(11)}}} -1 \rangle ^2 ) - 1]}\nonumber \\{} & {} + \displaystyle {\frac{a_{{\textrm{s}}}}{2b_{{\textrm{s}}}}} \displaystyle {[ \exp (b_{{\textrm{s}}} \langle {\bar{I}}_{{{4(22)}}} -1 \rangle ^2 ) - 1]}\nonumber \\{} & {} + \displaystyle {\frac{a_{{\textrm{n}}}}{2b_{{\textrm{n}}}}} \displaystyle {[ \exp (b_{{\textrm{n}}} \langle {\bar{I}}_{{{4(33)}}} -1 \rangle ^2 ) - 1]}\nonumber \\{} & {} + \displaystyle {\frac{a_{{\textrm{fs}}}}{2b_{{\textrm{fs}}}}} \displaystyle {[ \exp ( b_{{\textrm{fs}}} [{\bar{I}}_{{{4(12)}}} ]^2) - 1]}\nonumber \\{} & {} + \displaystyle {\frac{a_{{\textrm{sn}}}}{2b_{{\textrm{sn}}}}} \displaystyle {[ \exp (b_{{\textrm{sn}}} [ {\bar{I}}_{{{4(23)}}} ]^2 ) - 1]}. \end{aligned}$$

A combined triaxial-biaxial training of this model calibrates the model parameters to $a = 0.950$ kPa, $b = 5.457$, $a_{{\textrm{f}}} = 3.318$ kPa, $b_{{\textrm{f}}} = 23.701$, $a_{{\textrm{s}}} = 1.405$ kPa, $b_{{\textrm{s}}} = 20.067$, $a_{{\textrm{n}}} = 2.037$ kPa, $b_{{\textrm{n}}} = 16.976$, $a_{{\textrm{fs}}} = 0.586$ kPa, $b_{{\textrm{fs}}} = 1.081$, $a_{{\textrm{sn}}} = 0.047$ kPa, and $b_{{\textrm{sn}}} = 11.842$. This model has a mean goodness of fit $\textrm{R}^2 = 0.876$ [29]. We translate this constitutive model into our universal material subroutine through the definition of the following parameter lines in our finite element analysis input file

$$\begin{aligned} \begin{array}{l} {{{\texttt {*PARAMETER \,TABLE, TYPE={\text{"}}UNIVERSAL\_TAB{\text{"}}}}}} \\ \begin{array}{ccccccc} 1,&{}1,&{}1,&{}2,&{}1.0,&{}b,&{} a/2b \\ 4,&{}2,&{}2,&{}2,&{}1.0,&{}b_f,&{} a_f/2b_f \\ 8,&{}2,&{}2,&{}2,&{}1.0,&{}b_s,&{} a_s/2b_s \\ 14,&{}2,&{}2,&{}2,&{}1.0,&{}b_n,&{} a_n/2b_n \\ 6,&{}1,&{}2,&{}2,&{}1.0,&{}b_{fs},&{} a_{fs}/2b_{fs} \\ 12,&{}1,&{}2,&{}2,&{}1.0,&{}b_{sn},&{} a_{sn}/2b_{sn} \\ \end{array} \end{array}  \end{aligned}$$

Finally, we leverage an orthotropic constitutive neural network to discover the best model and parameters to explain the experimental data. From a library of $2^{32}=4,294,967,296$ possible combinations of terms and a sparsity-promoting regularization with $\alpha =0.01$, we discover a four-term model,

41 $$\begin{aligned} \displaystyle {\psi }&= {} {\mu ({\bar{I}}_2 - 3)^2 + \frac{a_{{\textrm{f}}}}{2b_{{\textrm{f}}}}[ \exp (b_{{\textrm{f}}} \langle {\bar{I}}_{{\mathrm{4(11)}}} -1 \rangle ^2 ) - 1 ]} \nonumber \\{} & {} + \frac{a_{{\textrm{n}}}}{2b_{{\textrm{n}}}} [ \exp (b_{{\textrm{n}}} \langle {\bar{I}}_{{\mathrm{4(33)}}} -1 \rangle ^2 ) - 1] \nonumber \\{} & {} +\frac{a_{{\textrm{fs}}}}{2b_{{\textrm{fs}}}} [ \exp ( b_{{\textrm{fs}}} [ {\bar{I}}_{{{4(12)}}} ]^2) - 1]. \end{aligned}$$

with a mean goodness of fit $\textrm{R}^2 = 0.894$ [29]. Here, our discovered material parameters amount to $\mu = 5.162$ kPa, $a_{{\textrm{f}}} = 3.426$ kPa, $b_{{\textrm{f}}} = 21.151$, $a_{{\textrm{n}}} = 2.754$ kPa, $b_{{\textrm{n}}} = 4.371$, $a_{{\textrm{fs}}} = 0.494$ kPa, and $b_{{\textrm{fs}}} = 0.508$. We integrate this newly discovered model for myocardial tissue in our finite element analysis through the following four-line parameter table

$$\begin{aligned} \begin{array}{l} {{{\texttt {*PARAMETER\, TABLE, TYPE={\text{"}}UNIVERSAL\_TAB{\text{"}}}}}} \\ \begin{array}{ccccccc} 2,&{}1,&{}2,&{}1,&{}1.0,&{}1.0,&{} \mu /2 \\ 4,&{}2,&{}2,&{}2,&{}1.0,&{}b_f,&{} a_f/2b_f \\ 14,&{}2,&{}2,&{}2,&{}1.0,&{}b_n,&{} a_n/2b_n \\ 6,&{}1,&{}2,&{}2,&{}1.0,&{}b_{fs},&{} a_{fs}/2b_{fs} \end{array} \end{array}  \end{aligned}$$

**Simulation**

We incorporate all three constitutive models for myocardial tissue in the finite element analysis software solver Abaqus [7] using our universal material subroutine, and predict the stress state of the left and right ventricular wall during diastolic filling. We create a finite element model of the left and right ventricular myocardial wall from high-resolution magnetic resonance images of a healthy 44-year-old Caucasian male with a height of 178 cm and weight of 70 kg [83, 84]. We spatially discretize our computational domain using 99,286 quadratic tetrahedral elements and 154,166 nodes, leading to a total 462,498 degrees of freedom. We compute the helically wrapped myofibers by solving a Laplace-Dirichlet problem across our computational domain, and assume a transmural fiber variation from +60$^{\circ }$ to $-60 ^{\circ }$ from the endocardial to the epicardial wall [117]. The resulting microstructural organization covers 99,286 local element-based fiber, sheet, and normal vectors, $\varvec{f}_0$, $\varvec{s}_0$, $\varvec{n}_0$. We apply homogeneous Dirichlet boundary conditions at the mitral, aortic, tricupid, and pulmonary valve annuli to fix the heart in space [85], and load it with hemodynamic Neumann boundary conditions that correspond to the endocardial blood pressure during diastolic filling. Figure 7 showcases the resulting deformation and stress profiles in both ventricles in response to left and right ventricular pressures of 8 and 4 mmHg. In a row-to-row comparison of the short-axis views, we observe small differences between the deformation invariants and the maximum principal wall stresses of all three models, with larger values for our newly discovered model and the Guan model and smaller values for the generalized myocardial Holzapfel model. We can explain these differences by the varying constitutive goodness of fit of the three models. Moreover, we observe that our diastolic hemodynamic loading conditions enforce deformation and stress states that surpass the homogeneous tissue testing protocols of the triaxial shear and biaxial extension training data. This creates local regions of extrapolation beyond the initial training regime [29].

## Conclusion

In this work, we designed a universal constitutive modeling framework to predict the mechanical behavior of soft materials across a wide range of applications. We set up a modular material subroutine architecture which seamlessly integrates with a commercial FEA framework and can easily be generalized towards other non-linear FEA solvers. Doing so, our framework mitigates the risk for human error and streamlines the integration of newly discovered material models in their simulations, thus alleviating the users to perform lengthy algebraic derivations and extensive programming. Furthermore, our material subroutine serves as an excellent verification tool for more expert finite element software developers aiming to debug their own soft material models and finite element analysis implementations. We demonstrated the versatility of the universal material subroutine through numerical simulations of various living systems including the brain, skins, arteries, valves and the human heart. Providing a common language and material subroutine for the computational mechanics community at large, we aspire to democratize the computational analysis of soft materials amongst a broader cohort of researchers and engineers. With one single subroutine, everyone - and not just a small group of expert specialists - can now perform reliable engineering analysis of artificial organs, stretchable electronics, soft robotics, smart textiles, and even artificial meat. Fostering this inclusivity, our framework can form an invaluable tool towards continued innovation and discovery in the field of soft matter overall.

## Figures and Tables

**Fig. 8:**
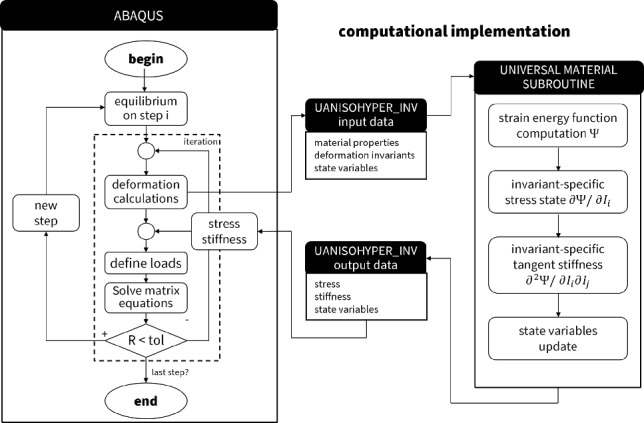
**Interaction between the finite element analysis solver and the universal material subroutine**. Flowchart of the interaction between Abaqus and the UANISOHYPER_INV subroutine architecture which embeds our universal constitutive material model. During each Newton–Raphson iteration and at each Gauss integration point, the UANISOHYPER_INV subroutine computes the free energy function $\psi$ (= UA), its first derivatives with respect to the deformation invariants ${\partial \psi }/{\partial \bar{I}_i}$ (= UI1(NINV)), and its second derivatives with respect to the deformation invariants ${\partial ^2 \psi }/{\partial \bar{I}_i \partial \bar{I}_j}$ (=UI2(NINV$^{*}$(NINV+1)/2)) with respect to the scalar invariants $\bar{I}_i$, derived from the deformation gradient $\varvec{F}$. These quantities are used by Abaqus to compute the components of the Cauchy stress tensor and the material tangent stiffness tensor, to construct the element force vector and stiffness matrix, and to assemble the global righthand side vector and stiffness matrix. Abaqus then performs a Newton–Raphson iteration based on the residual between the internal and external forces, until it achieves convergence

**Algorithm 1:**
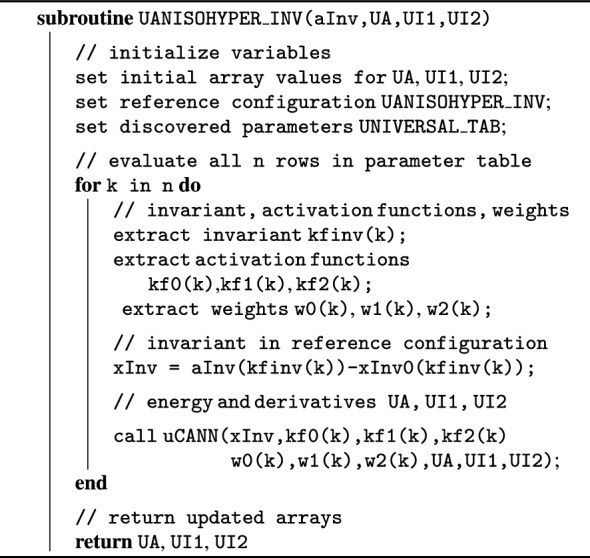
Pseudocode for universal material subroutine UANISOHYPER_INV

**Algorithm 2:**
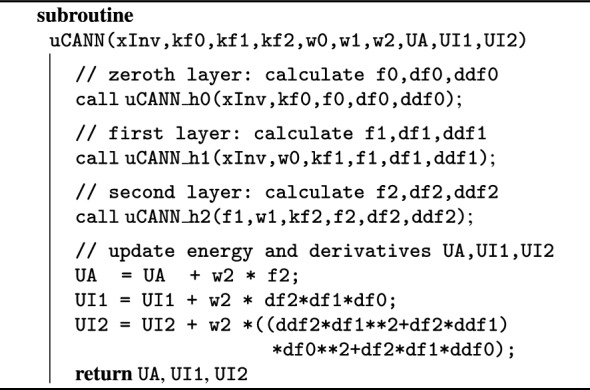
Pseudocode to update energy and its derivatives UA, UI1, UI2

**Algorithm 3:**
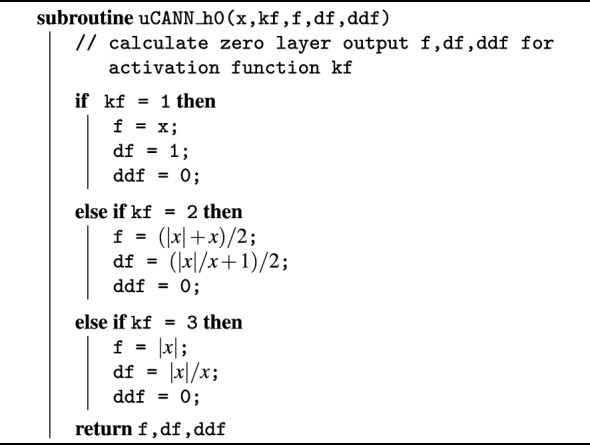
Pseudocode to evaluate output of zeroth network layer f,df,ddf

**Algorithm 4:**
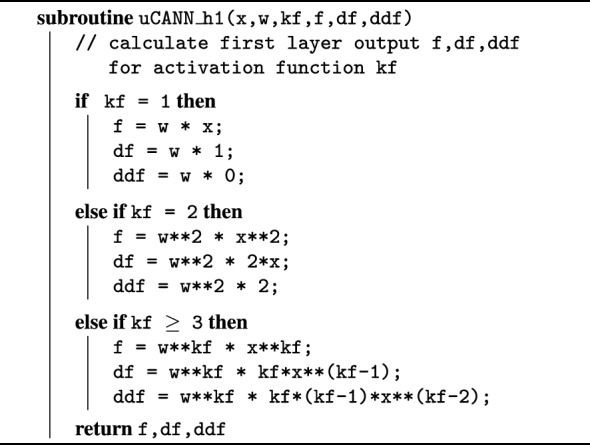
Pseudocode to evaluate output of first network layer f,df,ddf

**Algorithm 5:**
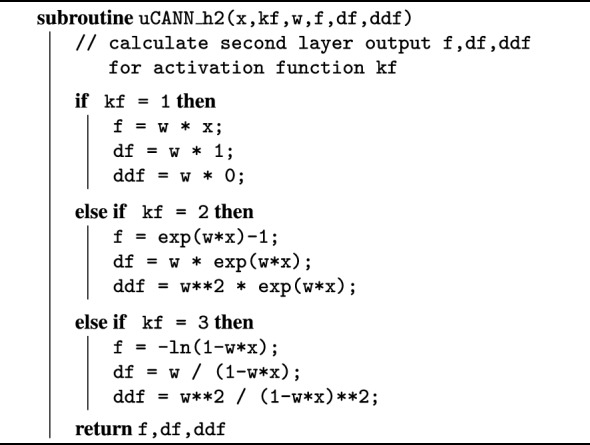
Pseudocode to evaluate output of second network layer f,df,ddf

## Data Availability

The code and simulation files are available at: github.com/peirlincklab/universalmatsubroutine.

## Appendix A: FEA integration

The concept of our universal material subroutine is inherently modular and generally compatible with any finite element analysis package. For illustrative purposes we implement our universal material model architecture in the Abaqus finite element analysis software suite. More specifically, we leverage the UANISOHYPER_INV user-defined subroutine architecture in Fig. 8 to seamlessly integrate our universal constitutive neural network architecture within the Abaqus FEA solver. This subroutine provides three input arrays: the constitutive model properties we provide through a constitutive parameter table in the finite element analysis input file; the deformation gradient invariants as defined in Eqs. (4), (5), (6), and (7); and an array of state-dependent field variables. Upon each evaluation, our user-defined subroutine *updates* the free energy function UA(1) $= {\bar{\psi }}$ and UA(2) $= \psi _{{\textrm{vol}}}$, the array of first derivatives of the free energy with respect to the scalar invariants UI1(NINV) $= {\partial \psi }/{\partial {\bar{I}}_i}$, and the array of second derivatives of the free energy with respect to the scalar invariants UI2(NINV)*(NINV+1)/2)$= {\partial ^2 \psi }/{\partial {\bar{I}}_i \partial {\bar{I}}_j}$. We detail these computations through the pseudocodes provided in Appendix E. To enable our UANISOHYPER_INV subroutine to read in a constitutive parameter table, we declare the format of this input parameter table using the parameter table type definition in the UNIVERSAL_PARAM_TYPES.INC file. This file reads

$$\begin{aligned} \begin{array}{l} {{{\texttt {*PARAMETER \,TABLE\,TYPE, name={\text{"}}UNIVERSAL\_TAB{\text{"}},}}}} \\  {{{\texttt {parameters = 7}}}} \\ \begin{array}{lll} {\texttt {INTEGER,}} &{}\,{\texttt {,}}&{}{{\texttt {{\text{"}}index \,invariant, kfinv,o{\text{"}}}}} \\ {\texttt {INTEGER,}} &{}\,{\texttt {,}}&{}{{\texttt {{\text{"}}index\, 0th \,activ\, function, kf0,o{\text{"}}}}} \\ {\texttt {INTEGER,}} &{}\,{\texttt {,}}&{}{{\texttt {{\text{"}}index \,1st \,activ\, function, kf1,o{\text{"}}}}} \\ {\texttt {INTEGER,}} &{}\,{\texttt {,}}&{}{{\texttt {{\text{"}}index \,2nd \,activ \,function, kf2,o{\text{"}}}}} \\ {\texttt {FLOAT,}} &{}\,{\texttt {,}}&{}{{\texttt {{\text{"}}weight\, 0th \,hidden\, layer, w0,o{\text{"}}}}} \\ {\texttt {FLOAT,}} &{}\,{\texttt {,}}&{}{{\texttt {{\text{"}}weight\, 1st\, hidden \,layer, w1,o{\text{"}}}}} \\ {\texttt {FLOAT,}} &{}\,{\texttt {,}}&{}{{\texttt {{\text{"}}weight\, 2nd\, hidden \,layer, w2,o{\text{"}}}}} \\ \end{array} \end{array}  \end{aligned}$$

and is introduced using the call

$$\begin{aligned} \begin{array}{l} {{{\texttt {*INCLUDE, INPUT=UNIVERSAL\_PARAM\_TYPES.INC}}}} \end{array}  \end{aligned}$$

at the start of our Abaqus FEA input files.

## Appendix B: FEA interface

To activate the universal material subroutine within our finite element analysis input file, we need to call our user-defined material model. When we incorporate a fully incompressible constitutive material model (i.e. including no $I_3$ invariant contributions), we call our user-defined material model through the command

$$\begin{aligned} \begin{array}{l} {{{\texttt {*ANISOTROPIC\;HYPERELASTIC,\,USER,\,FORMULATION=INVARIANT,}}}} \\ {{{\texttt {TYPE=INCOMPRESSIBLE,\,LOCAL\,DIRECTIONS=NDIR}}}} \end{array}  \end{aligned}$$

where integer NDIR defines the number of local fiber directions in our material. This input file command is followed up with the constitutive parameter table definitions described in Sect. 3.2. In contrast, when our free energy function contains a volumetric free energy $\psi _{{\textrm{vol}}}$ contribution, our universal material subroutine needs to additionally introduce UA(2), UI1(3) and UI2(6) (following the NINV$=3$ invariant numbering scheme in Eq. (15)) during its evaluations. To incorporate compressible material behavior in our FEA simulation, we change the TYPE keyword argument line in our Abaqus input file to TYPE = COMPRESSIBLE,

$$\begin{aligned} \begin{array}{l} {{{\texttt {*ANISOTROPIC\;HYPERELASTIC,\,USER,\,FORMULATION=INVARIANT,}}}} \\ {{{\texttt {TYPE=COMPRESSIBLE, LOCAL \,DIRECTIONS=NDIR}}}} \end{array}  \end{aligned}$$

where integer NDIR defines the number of local fiber directions of our material. Similar to above, this input file command is followed up with the constitutive parameter table definitions described in Sect. 3.2.

Importantly, our universal material subroutine computes the instantaneous elastic stress and stiffness response and allows a modular integration with other inelastic material behaviors that are commonly supported in commercial finite element analysis codes. For instance, in Abaqus the user can leverage the universal material model in conjunction with linear viscoelasticity to model the relaxation behavior of soft matter materials, and with damage or Mullins effect to account for stress softening in soft tissues. Additional combinations with plasticity, nonlinear viscoelasticity or creep, and more general damage models are also supported.

## Appendix C: Volumetric free energy functions

To model compressible material behavior using our modular material subroutine, we add the volumetric contributions to our constitutive parameter table along with all the other deviatoric free energy contributions,

$$\begin{aligned} \begin{array}{l} {{{\texttt {*\,PARAMETER\, TABLE, TYPE={\text{"}}UNIVERSAL\_TAB{\text{"}}}}}} \\ \begin{array}{ccccccc} \vdots &{}\vdots &{}\vdots &{}\vdots &{}\vdots &{}\vdots &{} \vdots \\ 3,&{}1,&{}1,&{}1,&{}1.0,&{}w_{1,\circ },&{} w_{2,\circ } \\ 3,&{}1,&{}2,&{}1,&{}1.0,&{}w_{1,\circ },&{} w_{2,\circ } \end{array} \end{array}  \end{aligned}$$

For example, the volumetric free energy function [118]

42 $$\begin{aligned} \psi _{\textrm{vol}}=\frac{K}{2}(I_3 - 1)^2 \end{aligned}$$

translates into the following contribution to the input file

$$\begin{aligned} \begin{array}{l} {{{\texttt {*PARAMETER\, TABLE, TYPE={\text{"}}UNIVERSAL\_TAB{\text{"}}}}}} \\ \begin{array}{ccccccc} 3,&{}1,&{}2,&{}1,&{}1.0,&{}1.0,&{}K/2\end{array} \end{array}  \end{aligned}$$

Alternatively, the volumetric free energy function

43 $$\begin{aligned} \psi _{\textrm{vol}}=\frac{K}{2} \left( \frac{I_3^2 - 1}{2} - \ln (I_3) \right) \end{aligned}$$

which is a special case of the modified Ogden formulation [5], can be reformulated to

44 $$\begin{aligned} \psi _{\textrm{vol}} = \frac{K}{2}\left( \!(I_3-1) + \frac{1}{2}(I_3-1)^2 - \ln (1-(-1)(I_3-1)) \!\right) \end{aligned}$$

which translates into the following lines in the input file

$$\begin{aligned} \begin{array}{l} {{{\texttt {*PARAMETER\, TABLE, TYPE={\text{"}}UNIVERSAL\_TAB{\text{"}}}}}} \\ \begin{array}{ccccccc} 3,&{}1,&{}1,&{}1,&{}1.0,&{}1.0,&{}K/2 \\ 3,&{}1,&{}2,&{}1,&{}1.0,&{}0.5,&{}K/2 \\ 3,&{}1,&{}1,&{}3,&{}1.0,&{}\text {-}1.0,&{}K/2 \end{array} \end{array}  \end{aligned}$$

## Appendix D: Mixed-invariant free energy functions

It is straightforward to generalize our universal material model architecture towards mixed-invariant models. Specifically, we create these mixed invariants as parameter-weighted combinations of two or more individual invariants. To incorporate mixed invariants in our material subroutine, we create a second parameter table type,

$$\begin{aligned} \begin{array}{l} {{{\texttt {*PARAMETER\, TABLE\,TYPE, name={\text{"}}MIXED\_INV{\text{"}},}}}} \\  {{{\texttt {parameters = 16}}}} \\ \begin{array}{llll} {\texttt {INTEGER,}} &{}\,{\texttt {,}}&{}{{\texttt {{\text{"}}index \,\,n \,of\, mixed\, invariant,}}} &{}{\texttt {Kinv,o{\text{"}}}} \\ {\texttt {FLOAT,}} &{}\,{\texttt {,}}&{}{{\texttt {{\text{"}}coefficient\, 1st\, mixed\, invariant,}}} &{}{\texttt { K1,o{\text{"}}}} \\ {\texttt {FLOAT,}} &{}\,{\texttt {,}}&{}{{\texttt {{\text{"}}coefficient\, 2nd\, mixed\, invariant,}}} &{}{\texttt { K2,o{\text{"}}}} \\ {\texttt {FLOAT,}} &{}\,{\texttt {,}}&{}{{\texttt {{\text{"}}coefficient \,3rd\, mixed\, invariant,}}} &{}{\texttt { K3,o{\text{"}}}} \\ \vdots &{} &{} \vdots &{}\vdots \\ {\texttt {FLOAT,}} &{}\,{\texttt {,}}&{}{{\texttt {{\text{"}}coefficient \,15th\, mixed\, invariant,}}} &{}{\texttt { K15,o{\text{"}}}} \end{array} \end{array}  \end{aligned}$$

where each of the 15 $\kappa _{j,\circ }$ mixed invariant coefficients denotes contributions to the mixed invariant $I_{\kappa ,\circ }$,

45 $$\begin{aligned} {\bar{I}}_{\kappa ,\circ }=\sum _{j=1}^{15} \kappa _{j,\circ } \, {\bar{I}}_j \end{aligned}$$

in which $\bar{I}_j$ follows the invariant numbering in Eq. (15). To activate these mixed invariants in our FEA model, we include the following lines in our FEA input file,

$$\begin{aligned} \begin{array}{l} {{{\texttt {*PARAMETER\, TABLE, TYPE={\text{"}}MIXED\_INV{\text{"}}}}}} \\ \begin{array}{llllllllll} 1,&{}\kappa _{1,1},&{}\kappa _{2,1},&{}\kappa _{3,1},&{}\kappa _{4,1},&{}\kappa _{5,1},&{}\kappa _{6,1},&{}\kappa _{7,1},&{}\kappa _{8,1},&{}\kappa _{9,1} \\ &{}\kappa _{10,1},&{}\kappa _{11,1},&{}\kappa _{12,1},&{}\kappa _{13,1},&{}\kappa _{14,1},&{}\kappa _{15,1}&{}&{} \\ 2,&{}\kappa _{1,2},&{}\kappa _{2,2},&{}\kappa _{3,2},&{}\kappa _{4,2},&{}\kappa _{5,2},&{}\kappa _{6,2},&{}\kappa _{7,2},&{}\kappa _{8,2},&{}\kappa _{9,2} \\ &{}\kappa _{10,2},&{}\kappa _{11,2},&{}\kappa _{12,2},&{}\kappa _{13,2},&{}\kappa _{14,2},&{}\kappa _{15,2}&{}&{}\\ \hdots \end{array} \end{array}  \end{aligned}$$

We activate any novel constitutive neuron associated with these mixed invariants, using the following argument lines in our FEA input file,

$$\begin{aligned} \begin{array}{l} {{{\texttt {*PARAMETER\, TABLE, TYPE={\text{"}}UNIVERSAL\_TAB{\text{"}}}}}} \\ 101,\texttt {kf0}_{\texttt {101}},\texttt {kf1}_{\texttt {101}},\texttt {kf2}_{\texttt {101}},\texttt{w}_{0,101},\texttt{w}_{1,101}, \texttt{w}_{1,101}\\ 102,\texttt {kf0}_{\texttt {102}},\texttt {kf1}_{\texttt {102}},\texttt {kf2}_{\texttt {102}},\texttt{w}_{0,102},\texttt{w}_{1,102}, \texttt{w}_{1,102} \\ \hdots \end{array}  \end{aligned}$$

To avoid any potential confusion between the standard invariants and the mixed invariants, we number all derived mixed invariants starting at NINV$=101$.

## Appendix E: Pseudocodes

In the following five algorithmic boxes, we summarize our universal material subroutine as pseudocode. Algorithm 1 illustrates the UANISOHYPER_INV pseudocode to compute the arrays UA(1), UA(2), UI1(NINV), and UI2(NINV $^*$(NINV+1)/2) at the integration point level. First, we initialize all relevant arrays and read the activation functions $kf_{0,k}$, $kf_{1,k}$ and $kf_{2,k}$ and weights $w_{0,k}$, $w_{1,k}$ and $w_{2,k}$ of the *n* constitutive neurons of our constitutive neural network from our user-defined parameter table UNIVERSAL_TAB (see Appendix B). Then, for each node, we evaluate its row in the parameter table UNIVERSAL_TAB and additively update the free energy density function and its first and second derivatives, UA, UI1, UI2. Algorithm 2 summarizes the additive update of the free energy and its first and second derivatives, UA, UI1, UI2, within the universal material subroutine uCANN. Algorithms 3, 4 and 5 provide the pseudocode for the three subroutines uCANN_h0, uCANN_h1 and uCANN_h2 that evaluate the zeroth, first and second network layers for each network node with its discovered activation functions and weights.
